# Disrupting membranes, controlling cell fate: the role of pore-forming proteins in cell death and therapy

**DOI:** 10.1007/s10495-025-02133-w

**Published:** 2025-07-21

**Authors:** Sonia Iranpour, Maryam Arif, Eva Szegezdi

**Affiliations:** 1 School of Biological and Chemical Sciences, University of Galway, Galway, Ireland; 2 Research Ireland Centre for Medical Devices (CÚRAM), Biomedical Sciences Building, University of Galway, Galway, Ireland

**Keywords:** Pore-forming proteins (PFP), Regulated cell death, Cancer therapy, Perforin, Granulysin, Gasdermin, Ninjurin-1, Mixed lineage kinase domain-like pseudokinase (MLKL)

## Abstract

Pore-forming proteins (PFPs), characterized by their ability to form pores or disrupt membranes are now recognized as key executioners of cell death, either as effectors of the immune system (non-cell-autonomous function), or of regulated cell death programs (cell autonomous function). To perforate membranes, most PFPs transition from water-soluble monomers or oligomers into multimeric and often supramolecular complexes, a process achieved via substantial structural transition of the PFP. Although they share the general ability to perforate cellular or intracellular membranes, PFPs differ in their membrane-binding preferences, the structural and functional characteristics of the pores they form (such as pore size, pore structure and ability to trigger membrane rupture) and the cell death mechanism they induce or execute. Herein, we review the specific traits of all key human PFPs, including their membrane specificity, regulation of their activity and the structure of the membrane pores they form, followed by insights into the therapeutic potential of PFPs and harnessing their abilities for cancer therapy.

## Introduction

Pore-forming proteins (PFPs) are a diverse group of proteins which permeabilize cellular membranes. Generally, PFPs are characterized by their ability to switch from a water-soluble form to a membrane-associated form and to arrange into pores. By forming of non-specific transmembrane pores or disrupting membrane integrity, they allow uncontrolled flux of ions and molecules across the membrane, disrupting osmotic balance, cell stability, and cellular homeostasis, ultimately leading to cell lysis [1]. Cell lysis by PFPs is an evolutionarily conserved mechanism across all kingdoms of organisms. For example, bacteria use PFPs (also called, pore-forming toxins, PFTs) to lyse host cells for the purpose of obtaining nutrients (e.g. red blood cell lysis) or to weaken the antibacterial immune response (e.g. by lysing neutrophils and other immune cells).

PFTs initiate membrane permeabilization by binding to specific cell membrane components, such as cholesterol and sphingolipids, cell surface receptors (e.g. b2-integrin, chemokine receptors) or glycans [2]. For example, perfringolysin O (PFO), a cholesterol-dependent cytolysin (CDC) [3, 4] secreted by the anaerobic bacterium, *Clostridium perfringens* [5] permeabilizes the membrane of endothelial cells, gut epithelial cells and neutrophil granulocytes by binding to cholesterol, while the RTX toxin, produced by *Bordetella pertussis* attacks membranes by binding to b2 integrin (CD11a/CD18 complex), highly expressed by leukocytes, thus impairing the immune response [6]. Binding to the membrane initiates a structural transition of the PFTs through which hydrophobic regions get exposed, facilitating their oligomerization. The PFT complex then inserts into the membrane, creating either small (2–4 nm in diameter) or large pores in the membrane (20–30 nm in diameter) [7]. Depending on the size, the pores allow free flux of ions or larger molecules through the membrane, causing osmotic imbalance or activation of programmed cell death pathways, ultimately causing cell death.

In mammals, instead of being virulence factors, PFPs function as immune effectors that lyse pathogens and damaged cells, or as intracellular mediators of cell death pathways [8–10]. The best-characterized group of mammalian PFPs is the membrane attack complex/perforin (MACPF) superfamily, defined by the MACPF domain, including the complement system components C6, C7, C8α/β, and C9 and the perforins, PRF1 and PRF2 (also known as MPEG1). Other mammalian PFPs include the gasdermin family (GSDMA–E and pejvakin/PJVK), the saposin-like protein, granulysin, mixed lineage kinase domain-like pseudokinase (MLKL) of the HeLo domain family, ninjurin-1 (NINJ1) [11] and a range of antimicrobial peptides, such as defensins and cathelicidin [12, 13]. Due to their diverse origins and evolutionary development, mammalian PFPs have traditionally been classified based on structural domains, such as the MACPF, HeLo, and GSDM domains, or their evolutionary relationships with bacterial counterparts, like CDCs. While these classification systems are well-known and have provided a framework for PFP classification, they fall short in capturing the functional similarities or convergence across PFPs belonging to different protein families. Therefore, in this review we also segregated PFPs into functionally-similar groups to complement the structural and evolutionary grouping. Based on function, we can distinguish secreted PFPs acting as inducers of non-autologous/non-self-lysis (i.e. PFPs acting on other cells) and intracellular PFPs, acting in a cell-autologous manner, i.e. permeabilizing the membrane of the cell expressing it (Table 1).

**Table 1:** Classification and functional characteristics of human pore-forming proteins (PFPs)

Type of PFP	Superfamily	Tissue origin	Type of cell death induced	Type of pore formed	Target cells	References
*Extracellular*						
Complement system PFPs: C6, C7, C8α/β, C9	MACPF	Hepatocytes, secreted into the blood	Necrosis	β-barrel pore	Microbes and damaged/aberrant host cells	[19]
PRF1	MACPF	Cytotoxic lymphocytes; stored in lytic granules; secreted upon antigen recognition into immune synapse	Apoptosis	β-barrel pore	Infected or aberrant host cells	[20]
Granulysin	Saposin-like proteins	Cytotoxic lymphocytes (CTLs and NK cells); stored in lytic granules, secreted upon antigen recognition into immune synapse	Microptosis	Carpet-like membrane disruption proposed	Bacteria, protozoa, fungi and parasitic cells, possibly host cell mitochondria	[21]
Defensins	Antimicrobial peptides, defensin family	Mostly innate immune cells; also, epithelial barrier cells (e.g. Paneth cells, mucosal epithelial cells), secreted upon microbial antigen recognition	Microbial cell lysis	Carpet and/or barrel stave pore proposed	Bacteria, fungi	[22]
Cathelicidin	Cathelicidin family (cathelin domain proteins)	Mostly innate immune cells (neutrophils, macrophages); epithelial barrier cells (e.g. mucosal epithelial cells, keratinocytes), secreted upon microbial antigen recognition	Microbial cell lysis	Carpet and/or barrel stave pore proposed	Bacteria, fungi, viruses	[23]
*Intracellular*						
PRF2	MACPF	Constitutively expressed in monocytic cells; inducible in barrier-forming cells (epithelial cells, fibroblasts)	Microbial cell lysis	β-barrel pore	Bacteria within phagosomes	[24]
GSDMs	Gasdermin	Broad expression; activated in immune cells upon inflammasome activation	Pyroptosis	β-barrel pore	Host plasma membrane and intracellular organelles	[25]
NINJ1	Ninjurin	Various tissues; upregulated during inflammation	Ferroptosis, necroptosis, pyroptosis, apoptosis	Non-classical pore (membrane rupture)	Host plasma membrane	[26]
MLKL	HeLo domain proteins	Expressed in various cell types	Necroptosis	Ion channel	Host plasma membrane	[27]

In this context, the group of secreted PFPs include the PFPs of the complement system, defensins, cathelicidin, granulysin and PRF1. Complement proteins, defensins, cathelicidin and granulysin form pores in the membranes of invading pathogens [14], while PRF1 targets self, somatic cells identified by effector immune cells as damaged or dangerous (e.g. infected or malignantly transformed).

Intracellular PFPs, typically functioning as executioners of cell death pathways, include the classical apoptosis mediators, Bcl-2-associated X protein (BAX) and BCL2-antagonist/killer 1 (BAK) [15], which form large pores in the mitochondrial outer membrane, followed by GSDMs [16, 17], MLKL [18] and NINJ1 [11], permeabilizing the plasma membrane during pyroptosis, necroptosis and ferroptosis.

The unique biological properties of PFPs, particularly their direct and potent cytotoxic activity against pathogens and abnormal hosts cells, makes them promising candidates for therapeutic development. For example, PFPs that target microbial pathogens hold substantial potential against multidrug resistant bacteria, while PFPs inducing host cell death can be developed into novel therapies to treat cancers unresponsive to conventional therapies. Although safe and targeted delivery of PFPs was a major challenge in the past, recent advances in nanotechnology and smart delivery systems now offer solutions to this barrier. This review summarizes these PFPs with a particular emphasis on their structural characteristics and mechanisms of action, followed by a discussion of their therapeutic potential against cancer to inspire further investigations into the intricate biology of PFPs.

PFP: Pore-forming protein; PRF: Perforin; MACPF: Membrane attack complex/perforin domain; CTLs: Cytotoxic T lymphocytes; NK cells: Natural killer cells; GSDM: Gasdermin; NINJ1: Ninjurin-1; MLKL: Mixed lineage kinase domain-like pseudokinase.

## Secreted pore-forming proteins of the immune system

Secreted PFPs are either produced by the liver and released into the periphery as inactive precursors, such as the complement system proteins, or produced by immune cells and deployed onto target cells through granule secretion.

### Complement system proteins

The complement system is a component of the innate immune system. It consists of a network of soluble proteins, secreted by hepatocytes into the blood [28]. Complement proteins recognize pathogens via pathogen-associated molecular pattern (PAMP) molecules and also detect dead, stressed or malignant host cells [29] via their expression or release of damage-associated molecular patterns (DAMP) molecules [30, 31]. Upon recognition, they bind to these cells thus labelling them for destruction (process called opsonization) or directly lyse them.

The complement system can be activated through one of three routes: the classical pathway, the lectin-mediated pathway and the alternative pathway (for a comprehensive review of the activation and regulation of the complement system please refer to articles by Merle and colleagues [32, 33]). Regardless of the mechanism, activation of the complement system leads to the formation of the membrane-attack complex, or MAC [34]. The initiating step of MAC assembly is the activation of the complement protein, C5 by its cleavage into 2 fragments: C5a and C5b by the C5 convertase serine protease complex. The produced C5b fragment covalently anchors itself to the membrane of the pathogen via its thioester domain [35] and recruits the complement protein C6, followed by C7. The MACPF domain of C7 submerges into the membrane, stably linking the complement complex with the surface of the pathogen. The insertion of C7 into the pathogen’s membrane is a critical step that enhances the stability of the MAC and allows for the recruitment of C8 [36, 37]. C8 is a heterotrimeric complex, consisting of C8α, C8β and Cγ. C8α and C8β also insert their hydrophobic MACPF domains in the target cell’s membrane, further stabilizing the complex [38]. The thus formed C5b-8 complex serves as a membrane receptor for the last MACPF-containing complement component, C9 and nucleates the transmembrane pore. It sequentially recruits approximately 16–18 individual C9 molecules propagating pore growth in a unidirectional, clockwise manner during which the MACPF domains of the arriving C9 units form a barrel-stave pore, similar to that formed by PRF1 and PRF2 (see Sects. ”Perforin-1” and “The antibacterial perforin: Perforin-2”).

C6, C7, C8α, C8β and C9 all have a MACPF domain composed of a central, twisted β-sheet arranged in an anti-parallel fashion, surrounded by two groups of α-helices (CH1 and CH2, which are functionally equivalent to the transmembrane hairpin (TMH)-1 and -2 regions of PRF1 and 2). Importantly, the CH1 and CH2 regions within the MACPF domains of C6, C7, C8α, C8β differ notably from those of C9 in their hydrophilicity and length. For instance, the CH segments of C7 are less hydrophobic and shorter than of C9 (CH1: 44 amino acids (aa) vs 70, CH2: 47 aa vs 57, for C7 and C9, respectively), likely preventing C7 from fully spanning the membrane and explaining why C9 units form the staves of the transmembrane pore [36, 39, 40].

The fully-formed pore has an asymmetrical, split-washer shape, consisting of three regions: an asymmetric stalk region (C5b8), a hinge region (C7, C8, and two C9), forming 4 staves of the barrel-stave pore not fully spanning the membrane, and a C9 oligomer (16–18 units) [38] forming a ring of 16–18 membrane-spanning staves as the main body of the MAC pore [37]. The complement pore can vary in size and degrees of membrane insertion and distortion [41] and the MAC pore ring displays can function either in an open ring or closed ring conformation [34]. In the open form, the asymmetric stalk region does not connect with the last C9 unit, leaving a chasm (a 30-Å wide gap) along the side of the ring running through the full length of the pore. In this form, the stalk appears as a paddle or flap protruding into the lumen of the pore when viewed from the top. To adapt the closed ring conformation, the asymmetric C5b-8 paddle swings towards and connects to the last C9 unit, sealing the chasm on the side of the pore. This plasticity of the complement MAC pore has been suggested to enhance membrane destabilization in pathogens with tightly packed membranes (e.g., bacterial membranes enriched in polysaccharides and porins) [34, 37].

Through the MAC pore, ions can pass, disrupting normal ion gradients and inducing cell lysis [42]. The MAC complex typically lyses Gram-negative bacteria, which is counterintuitive, considering that Gram-negative bacteria have a double-layered membrane structure, consisting of an outer membrane and an inner membrane with a gelatinous matrix of peptidoglycans in between. Although the MAC pore can only span one of these membrane layers, it has been shown that the outer membrane stress is sufficient to destabilize the inner membrane [43]. Disruption of the outer membrane tears the protein connections between the outer and inner membrane, which reduces envelope stability [44]. It has also been proposed that the cellular stress induced by outer membrane permeabilization activates a bacterial programmed cell death machinery, for example via a toxin-antitoxin system (TA) [45]. Alternatively, the host-secreted glycoside hydrolase enzyme, lysozyme (present in the blood) enters into the periplasmic space via the MAC pores where it degrades the gel-like matrix of peptidoglycans and thus exposes the inner, cytoplasmic membrane to complement attack [46].

To the best of our knowledge, the MAC does not possess any known or specific membrane-binding domain or target, enabling its assembly on a wide variety of membranes [34]. However, several regulatory factors operating at different levels ensure that perforation is directed at abnormal host cells or microbes. Firstly, activation of MAC formation is targeted, induced locally, adjacent to the invading pathogens, where identification of pathogens through bacteria-expressed PAMPs initiates the tagging of the bacterial surface by the C5 convertase, so the cleavage of C5 takes place on the microbial surface. Importantly, effective MAC-dependent killing is contingent upon the immediate anchoring of C5b7 complexes to the target membrane [47]. This is because the soluble C5b7 pre-complex (sC5b7) is inherently unstable, lytically inactive and susceptible to scavenging by chaperones, such as clusterin, S protein and soluble-phase C8 (non-membrane-associated C8). These interactions prevent the insertion of sC5b7 into non-targeted lipid bilayers, thereby averting unintended membrane perforation [48, 49].

Secondly, strong regulatory mechanisms are in place to mitigate accidental MAC activation on host cells. A key regulator of this is CD59, a glycosylphosphatidylinositol (GPI)-anchored surface protein present on the surface of most somatic cells, especially red and white blood cells. It has been proposed that CD59 is activated by the impact of MAC pore formation on membrane mechanoproperties (rigidifying) [34]. Once active, CD59 interacts with the C5b-8 complex, as well as the C5b8 complex bound to an initial C9 molecule and inhibits the recruitment of further C9 units. This prevents the formation of a functional MAC pore thereby protecting host cell integrity [50].

### Pore-forming proteins released by effector immune cells

The immune system employs diverse strategies to fight against infections while minimizing harm to healthy tissues. One such mechanism is the release of secretory vesicles or granules which contain cytotoxic proteins. For example, neutrophil granulocytes carry three distinct types of secretory granules, each with specialized cargo and function [51], while cytotoxic lymphocytes, including natural killer (NK) cells and CD8^+^ T cells, contain so-called lytic granules (LGs), which store cytotoxic proteins specialized for the killing of infected or damaged self-cells [52]. These specialized granules are exocytosed, releasing cytotoxic effector molecules directly onto target cells, ensuring targeted pathogen clearance or destruction of infected and abnormal cells. A critical component of these secretory vesicles is PFPs, most notably PRF1, granulysin and antimicrobial peptides (AMPs) [51, 53].

In this section, we provide a comprehensive overview of human PFPs within secretory and lytic granules, including PRF1, granulysin and the human AMPs, defensins and cathelicidin, highlighting their structural characteristics and mechanisms of membrane perforation in target cells.

#### Perforin-1

LG are frequently characterized as “secretory lysosomes” due to their dual nature. While they are secretory granules (unlike lysosomes), they also have characteristics similar to lysosomes, such as an acidic luminal pH and a hydrolytic enzyme cargo, such as cathepsins. Additionally, the membrane of LGs contains LAMP1 (CD107a), a lysosome membrane marker, further illustrating their dual, lysosome-like and secretory characteristics [54].

PFPs found in LGs include PRF1 and granulysin, stored together with 1) other cytotoxic proteins, such as granzymes (GZMs), 2) death ligands (Fas ligand (FasL), tumor necrosis factor (TNF)-related apoptosis inducing ligand (TRAIL)) and 3) proteins responsible for retaining PFPs and cytotoxic proteins in an inactive state, such as serglycin (SRGN) and calreticulin (CRT) [55–57].

Activation of the antigen receptor initiates the formation of the immune synapse (IS), an extensive molecular complex enabling stable attachment of the immune cell to the target cell thus targeting the cytotoxic attack towards the target cell. In the immune cell, the cytoskeleton is rearranged forming microtubules that stabilize the IS and to guide LGs toward the IS. LGs are then transported to the IS. When at the IS, LGs dock at the plasma membrane and undergo priming, a process that prepares them for exocytosis. Then, the LGs fuse with the plasma membrane, releasing their content, including PFPs, into the cleft between the immune cell and the target cell [58].

PRF1 is the best characterized PFP of LGs. It is a 70 kDa glycoprotein belonging to the of MACPF protein superfamily and expressed by cytotoxic T cells and NK cells. The protein has three conserved domains, starting with a MACPF domain at the amino terminus, followed by a middle, epidermal growth factor (EGF)-like domain and a Ca^2+^-binding and membrane docking, C2 domain at the carboxyl end [59].

PRF1 permeabilizes membranes of host (mammalian) cells. Membrane insertion is initiated by the C2 domain. Upon release from LGs into the immune synapse, the C2 domain binds Ca^2+^ ions [60], which alters its electrostatic state thus enabling the interaction of C2 with membrane phospholipids, which in turn initiates conformational changes in the MACPF domain. The MACPF domain comprises of a central β-sheet surrounded by three α-helical regions, known as TMH-1 and -2, and a helix-turn-helix (HTH) motif [59]. Upon membrane docking by the C2 domain, TMH-1 and -2 undergo a drastic structural change and from an α-helical, closed structure they transition into amphipathic β-hairpins and extend outward, exposing hydrophobic amino acids during the process. The extended TMHs submerge into the membrane of the target cell and form lateral interactions with other PRF1 monomers thus assembling into a giant β-barrel formed by 19–24 PRF1 units spanning the target cell membrane [59]. The function of the EGF domain is less well-understood. According to the current model, it has a flexible architecture, allowing it to be pulled by TMH2 during membrane insertion, thereby filling the gap previously occupied by TMH1. Through this repositioning, it is believed that the EGF domain stabilizes the oligomeric structure [61].

The activity of PRF1 is tightly controlled to prevent permeabilization of the immune cell’s own membranes during intracellular trafficking and storage of PRF1. Firstly, the endoplasmic reticulum (ER) and Golgi vesicles are protected from PRF1-mediated perforation by PRF1’s C-terminal tail. The C-terminal tail facilitates a fast transportation of PRF1 from the ER to the Golgi, thus minimizing intracellular membrane damage [62]. Furthermore, a posttranslational glycan modification of the C terminal tail (on asparagine in position 549 (Asn549)) has been shown to play a similar role. It has been shown it also enhances fast transportation of PRF1 and additionally, it blocks PRF1 oligomerization to prevent ER- and Golgi membrane damage [63]. Once in the LG, the C-terminus of PRF1 is trimmed off by proteases, such as cathepsin C, H and L [64]. The trimming appears to be non-specific, with several truncated forms of PRF1 present in LGs, with up to 15 amino acids removed from the C-terminus [63]. This C-terminal trimming also removes the Asn549 glycan, thus relieving the oligomerization block. Despite the loss of the C-terminal tail, PRF1 remains inactive in LGs due to the acidic luminal pH and low calcium concentration [65]. Furthermore, in LGs PRF1 is complexed with the proteoglycan, SRGN and the chaperone protein, CRT, also keeping PRF1 inactive [56, 57, 66].

SRGN contains long, negatively charged chains of sulfated glycosaminoglycans (GAGs), which interact with the positively charged residues of PRF1 at acidic pH, maintaining PRF1 in an inactive state until secretion [57, 67]. SRGN and PRF1 can form large protein complexes, called supramolecule attack particles (SMAPs), which are large (120–500 nm diameter) membraneless structures composed of a core of PRF1 and GZMB along with SRGN and galectin-1, encased in a glycoprotein shell of thrombospondin-1 (TSP-1). SMAPs do not only maintain PRF1 inactive, but owing to their long half-life time after secretion, they provide a mechanism for sustained PRF1 and GZM activity enabling killing of multiple target cells [68, 69]. Finally, in the absence of calcium ions, PRF1 forms a strong interaction with the P-domain of CRT, providing an additional layer of protection against premature PRF1 activation [70, 71].

Once PRF1 is secreted in the neutral pH and high-micromolar to millimolar Ca^2+^ levels in the IS, PRF1 dissociates from its inhibitory partners and the C2 domain binds Ca^2+^, activating its membrane-binding capability. Several key properties of target membranes, including lipid order, lipid headgroups, charge and membrane tension have been proposed to regulate membrane anchorage of PRF1 [72, 73]. Initial studies showed that phosphatidyl choline, one of the most abundant phospholipids in the mammalian plasma membrane may serve as the binding site for PRF1 [74], but later the emphasis shifted from the chemical identity of the polar head groups to the physicochemical properties of the membrane. These studies revealed that loose lipid spacing in membranes facilitates PRF1 pore formation, while tightly packed membranes, typical of cytotoxic lymphocytes prevent PRF1 insertion [75]. Accordingly, membranes rich in unsaturated acyl chains and adapting a liquid-disordered state are permeabilized by PRF1. On the contrary, solid-ordered phase membrane modules and the presence of negatively charged phospholipids (e.g. phosphatidyl serine; PS) prevent PRF1 binding and pore formation, respectively [76].

**Fig. 1:**
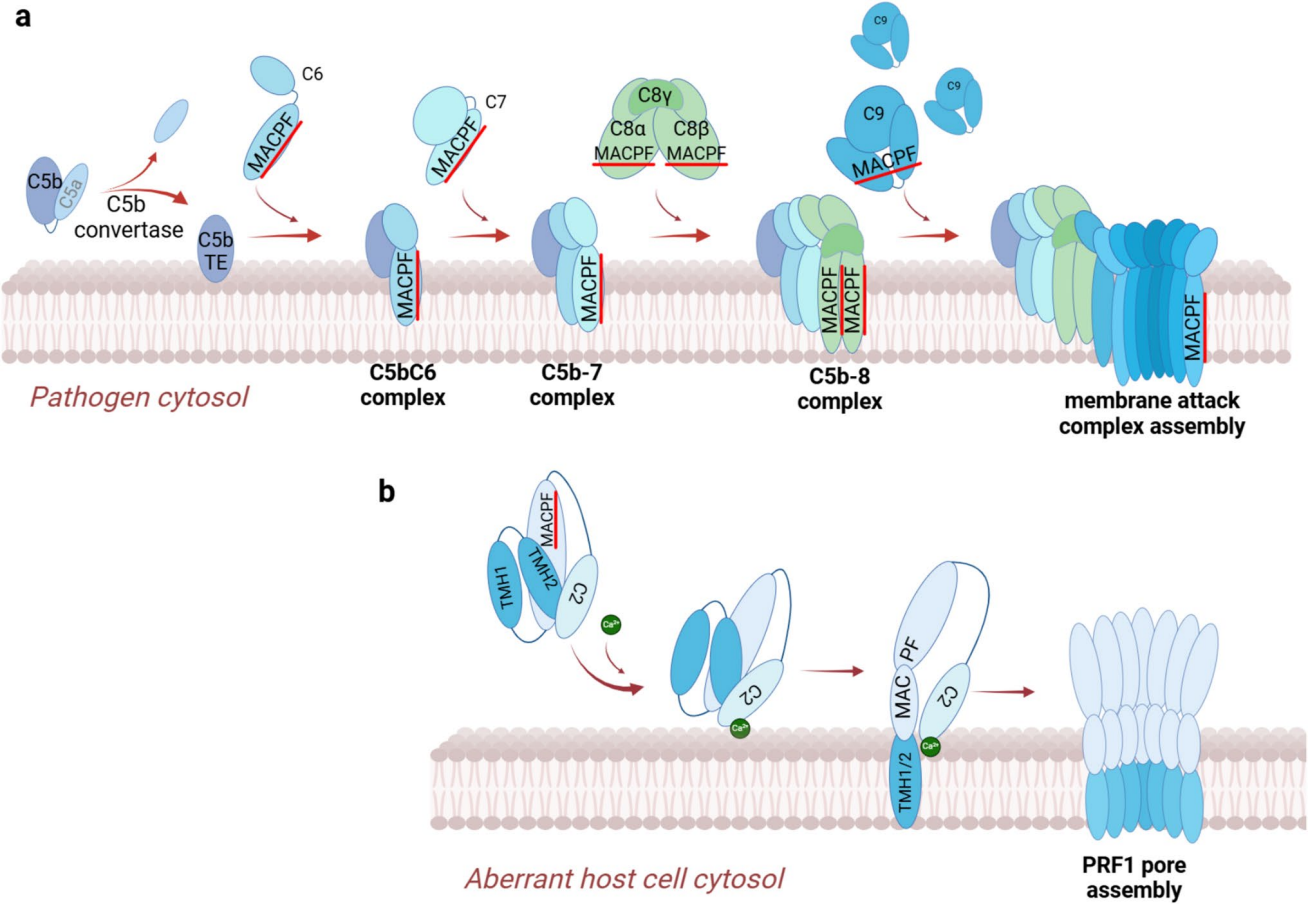
Formation of membrane attack complex pores by the complement system and perforin-1. A. Stepwise assembly of the complement MAC (membrane attack complex). The C5 convertase (not shown) cleaves and thus releases the C5b complement component, which then covalently binds the membrane via its thioester domain. C5b then interacts with C6, the first complement component carrying a membrane attack complex and perforin domain (MACPF). The C5b-C6 complex recruits C7, which undergoes a conformational change extending and immersing its MACPF domain in the membrane. Recruitment is C7 is followed by attachment of the C8 complex and extension and insertion of the C8a and C8b MACPF domains into the lipid bilayer. The thus assembled C5b-C8 complex recruits the first C9 unit and nucleates the complement MAC complex by recruiting 16–18 C9 units, whose MACPF domains form a split-washer shaped pore with the attached C5b-C8 stalk region. B. Formation of the perforin-1 (PRF1) membrane pore. The MACPF domain of inactive PRF1 is enveloped by two alpha-helical TMH domains. Upon release from lytic granules, the C2 domain of PRF1 binds Calcium ions (4–5 Ca^2+^ per domain) enabling it to bind to the plasma membrane of the target cell (host/mammalian cell). The lipid-C2 interaction initiates conformational changes in the TMH1 and PMH2 domains whereby their alpha-helical fold shifts into a long, extended beta-fold, which immerses and transpasses the target membrane. Several (18–22) PRF1 units oligomerize via lateral interactions to form the PRF1 membrane pores. Figure was generated with BioRender

PRF1 creates pores in the target cell membrane through a three-stage process, starting with membrane docking, followed by formation of the pre-pore intermediate state and finally the assembly of the mature pore [59]. In the first stage, the C2 domain forms co-ordinated links with up to five Ca^2+^ ions, which leads to its conformational change and enabling the interaction with phospholipid head groups [60]. Next, 2–5 PRF1 units oligomerize, forming pre-pores on the surface of the target membrane. These pre-pores can be categorized into two types: early and late, with the late pre-pores being larger and more tightly packed than the early-stage pre-pore structures [77]. Pre-pores then insert themselves into the membrane and serve as a nucleation site for the recruitment of additional pre-pore assemblies to the growing pore till 10–20 nm diameter ring- or arc-shaped mature transmembrane pores are generated [77] (Fig. 1b). The mature PRF1 pores are large enough to allow free flux of not only ions, but also of proteins, most notably the entry of the cytotoxic GZM enzymes into the target cells. The uncontrolled influx and efflux of ions disrupts cellular homeostasis by triggering a tonic shock, ultimately resulting in necrosis [78], while the entry of GZMs into the target cell’s cytoplasm results in apoptotic cell death.

Importantly, target cells can protect themselves from PRF1 attack by launching a membrane-repair response. The influx of Ca^2^⁺ from the extracellular space triggers a membrane-repair response, during which intracellular vesicles are recruited to the damage site and exocytosed in order to donate their membranes to reseal the damaged membrane [79]. At the same time, the damaged membrane sections are removed by endocytosis [80]. GZMB has been shown to stably bind to the outer surface of the membrane via mannose-6-phosphate receptors (MPR) and thus GZMB gets endocytosed together with the damaged, PRF1-containing membrane sections. The endocytosed membranes form enlarged endosomes (also called gigantosomes) and serve as an important, indirect pathway for the internalization of GZMB [79, 81]. It is proposed that after endocytosis, PRF1 can permeabilize and disrupt the gigantosome’s membrane, allowing the release of GZMs into the cytosol [82].

**Fig. 2:**
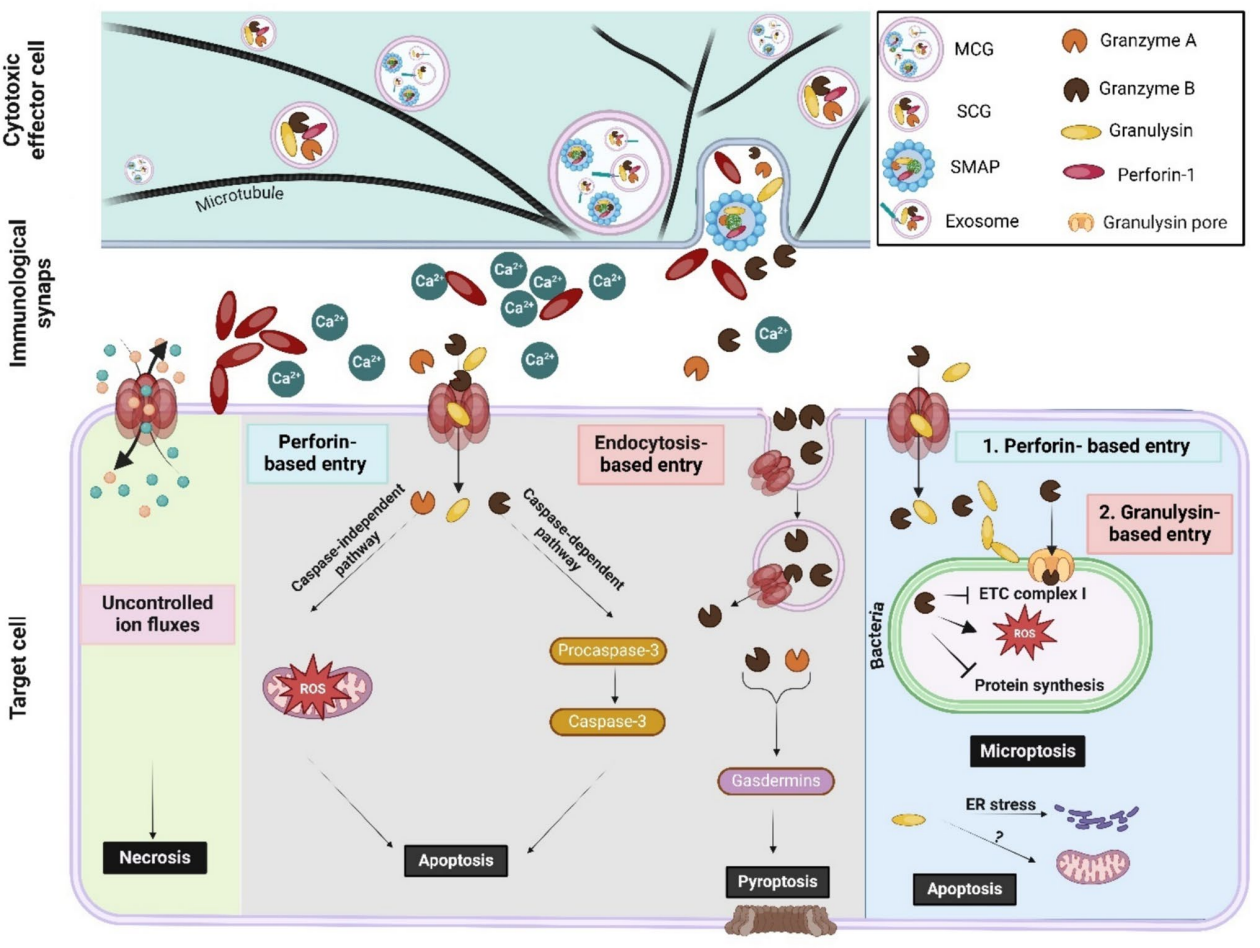
Schematic representation of cytotoxic mechanisms mediated by lytic granule pore-forming proteins. There are two distinct classes of lytic granules (LGs) based on their size, morphology, cargo, and function, namely single-core granules (SCGs) and multi-core granules (MCGs). Supramolecule attack particles (SMAPs) and exosomes accumulate in MCGs. Both types of granules are transported along microtubules to the immune synapse, where they undergo exocytosis to release their contents. Once released, perforin-1 (PRF1) is activated by the neutral extracellular pH and calcium ions (Ca^2+^) and forms transmembrane pores in the target cells. These pores allow uncontrolled ionic flux and if not repaired promptly, they result in necrosis of the target cell. PRF1 also enables the delivery of granzymes (GZMs) into target cells, either directly through the PRF1 pores or indirectly, through endocytosis of damaged membrane segments to which GZMB binds. Once in the cytosol, GZMs induce apoptotic cell death through caspase activation and by inducing oxidative stress by inactivating mitochondrial electron transport chain components (e.g. NDUFS3). GZMs also trigger pyroptotic cell death by cleaving and activating gasdermins (GSDMs). Granulysin also enters the target cell through PRF1 pores. Once in the cytosol, it ruptures the membranes of intracellular bacterial driving their lysis as well as facilitating the delivery of GZMs into the microbial cytoplasm. GZMs cleave electron transport chain proteins that triggers microptosis. Additionally, granulysin may also perforate the endoplasmic reticulum (ER) and promote ER stress and damage mitochondria, triggering apoptosis of infected self-cells. Figure was generated with BioRender

GZMs are a family of serine proteases, with five members identified in humans: A, B, H, K, and M. Each GZM targets specific substrates to induce cell death through distinct mechanisms. GZMA for instance, induces cell death through a caspase-independent pathway by cleaving NDUFS3 (NADH-Ubiquinone Oxidoreductase Core Subunit S3), a component of Complex I in the mitochondrial electron transport chain, leading to increased reactive oxygen species (ROS) production and oxidative stress [83]. GZMA can also cleave and activate GSDMB, which induces pyroptosis (discussed below, in Sect. ”Gasdermins”) [84]. GZMB, acting in parallel with GZMA, activates the classical apoptotic pathway through cleaving and activating pro-caspase-3 and the BH_3_-only Bcl-2 protein, Bid, into truncated Bid (tBid) (Fig. 2) [85]. Similar to GZMA, GZMB can also cleave and activate a gasdermin family member, GSDM, thereby inducing pyroptosis [86].

As discussed earlier, immune cells employ a variety of protective mechanisms to prevent membrane damage and cell lysis during the intracellular trafficking of PRF1. Protective mechanisms continue to function after the release of PRF1, shielding the immune cells from PRF1 toxicity. For example, upon degranulation, cathepsin B, which is bound to the outer membrane surface of lymphocytes, may provide protection by cleaving and thus inactivating PRF1 [87]. Of note, despite its protective function, cathepsin B appears to be insufficient in providing complete immunity against PRF1 activity [88]. Immune cells can also rearrange their membrane into a denser-packed, gel/solid-phase state through the incorporation of cholesterolto block PRF1 insertion. They can also externalize the negatively charged lipid, PS, to inactivate any PRF1 that may bind to the immune cell’s membrane [76, 89]. However, not all areas of the immune cell membrane are fully protected from PRF1 attack. Recently, it has been indicated that T cells mitigate this risk by reducing their cortical actin filaments through the downregulation of the cytoskeletal protein, filamin A (FLNA). FLNA plays a pivotal role in stabilizing the conformation of PRF1 during its insertion into the target cell membrane. Through this mechanism, cytotoxic T cells leverage membrane softness to prevent PRF1-mediated autolysis [90]. Overall, the combination of a range of resistance mechanisms to mitigate the cytotoxic effects of their own deadly cargos prevents self-harm of PRF1-secreting immune cells and ensures the unidirectional toxicity of PRF1.

#### Granulysin: a dual-function immune effector

Granulysin is another PFP of LGs. It belongs to the saposin-like protein family (SAPLIP) and it is composed of five α-helical bundles, stabilized by two intramolecular disulfide bonds [91]. Granulysin is synthesized as a 15 kDa precursor and transported to the LGs in this form. Within the LGs, it is cleaved by cathepsins at both termini to produce its 9 kDa, active form. Evidence indicates that granulysin is also stored in lymphocyte vesicles other than LGs. In these vesicles, granulysin does not get processed, instead, it is stored as the 15 kDa, full length protein. This isoform is not able to attack membranes and functions as an immune alarmin [92].

Mature granulysin (9 kDa) can interact with negatively charged phospholipids, such as phosphatidylglycerol and cardiolipin in membranes via its positively charged surfaces. Moreover, its activity is inhibited by cholesterol, thus making bacterial, protozoal, fungal, and parasitic cell membranes its preferred targets [93, 94].

The 9 kDa granulysin is inactive inside LGs, due to the acidic luminal pH and high cholesterol content [21]. After degranulation, granulysin becomes active and can directly attack the membrane of extracellular pathogens and can also enter infected host cells through PRF1 pores [95] and possibly by endocytosis at cholesterol-poor lipid rafts [93] and thus reach intracellular pathogens. In terms of intracellular pathogens, granulysin works in conjunction with PRF1 to deliver GZMs into the cytosol of microbes where the plasma membrane of the infected host cell is permeabilized by PRF1, through which granulysin and GZMs enter. Once inside, granulysin attacks the membrane of the intracellular pathogen, enabling entry of GZMs [96–98]. GZMs in the pathogen microbes cleave and thus inactivate components of the electron transport chain (e.g. complex I), which promotes ROS production, mitochondrial outer membrane permeabilization and protein synthesis block, which culminates in microptosis, a microbial programmed cell death (PCD) process [21].

While the molecular mechanism of granulysin pore formation is not completely resolved, it has been proposed that granulysin covers the microbial cell membrane in a carpet-like layer driven by electrostatic interactions. As granulysin clusters on the membrane’s surface, it interacts with the phospholipids leading to micelle formation thus removing lipids that generates gaps in the membrane [91]. Through these gaps GZMs enter into the microbes where it induces microptosis [97–99].

In addition to attacking microbes, granulysin has been reported to induce apoptotic cell death in host (mammalian) cells. It has been suggested that mitochondrial membranes, which are similar in composition to prokaryotic membranes, i.e. poor in cholesterol but rich in cardiolipin, may serve as targets for granulysin [21]. Notably, much of this research used the 9 kDa recombinant granulysin and detected mitochondrial damage, but these studies have faced challenges as bacterial-expressed, recombinant granulysin might not had the correct, natural folding. Additionally, in vivo, granulysin is present at a significantly lower concentrations in LGs than the concentrations of recombinant granulysin used (nM vs mM), which likely alters its interaction with target membranes [100–102]. Interestingly, further evaluation of granulysin-mediated toxicity using granulysin transgenic mice (since mice do not have a granulysin gene) revealed that granulysin predominantly targeted the endoplasmic reticulum (ER) and induced ER stress-mediated apoptosis. This finding suggests that granulysin may primarily exert its toxic effects on the ER rather than the mitochondria [95].

#### Antimicrobial peptides

AMPs are small proteins used by the innate immune system to kill bacteria, fungi and viruses. Additionally, AMPs modulate the immune response by attracting immune cells to infection sites and supporting wound healing. AMPs are produced across all biological phyla and as of 2024, the Antimicrobial Peptide Database has a record of 3146 natural AMPs [103].

**Fig. 3:**
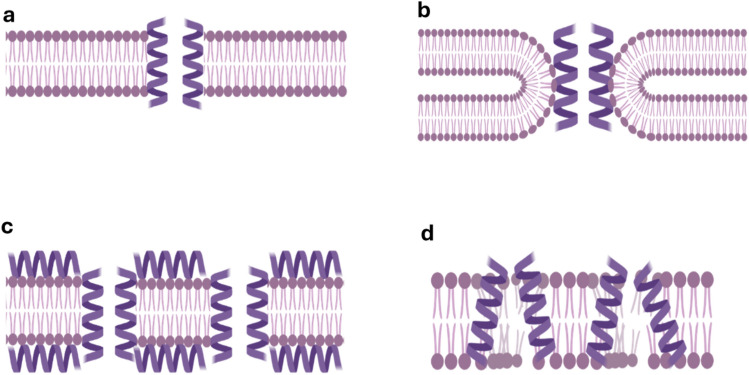
Illustration of the main mechanisms of pore formation and membrane disruption by antimicrobial peptides (AMP). a) The barrel-stave model. The AMPs insert into the membrane, forming water-filled pores lined by an antimicrobial peptide (AMP) oligomer exposing a hydrophilic face towards the lumen and a hydrophobic face towards the lipid bilayer. b) The toroidal-pore model. AMPs form a toroidal pore where the wall of the pore incorporates both AMP and lipid components, bending the membrane into a continuous toroidal structure. c) The carpet model. AMPs accumulate and cover the membrane surface where they interact with membrane lipids. The AMPs disrupt membrane continuity by removing lipids from it after forming AMP-lipid micelles in a detergent-like manner without forming discrete pores. d) The aggregate model. AMPs form transient peptide-lipid complexes, allowing ion leakage, removal of lipid micelles and facilitating intracellular protein entry. Figure was generated with BioRender

AMPs use a variety of mechanisms to kill microbes by membrane-permeabilization or immunomodulation [104]. Membrane-targeting AMPs tend to interact with negatively charged lipids and other components typical of microbial membranes resulting in increased membrane permeability and eventual cell lysis [104]. As a diverse group of proteins, AMPs employ various mechanisms to permeabilize membranes. These mechanisms can be broadly grouped into four models: the barrel-stave-, toroidal-, carpet- and the aggregate model [105]. In the barrel-stave model, the peptides initially bind to the membrane through electrostatic interactions. This leads to accumulation of the peptides on the membrane surface causing local lipid rearrangement and membrane thinning, allowing the AMPs to insert in the membrane and assemble into a barrel-like oligomeric pore [105, 106] where the hydrophobic regions of the peptides interact with the lipid bilayer, while the hydrophilic regions face inward, forming a central pore (Fig. 3a).

The toroidal-pore model is similar, but with both AMP peptides and lipids contributing to the pore. Here, interaction of the AMPs with the membrane forces the membrane to bend, which allows the AMPs to “cap” the lipids and form an AMP-lipid complex in the lipid bilayer which arranges into a toroidal pore (Fig. 3b).

In the carpet and aggregate models, instead of forming channels, AMPs accumulate on the membrane surface, parallel with the membrane surface [107]. In the carpet model, when the concentration of the peptides reaches a critical threshold, they disrupt the membrane by acting like detergents, displacing lipids from the membrane into micelles and causing the membrane to fragment into micelle-like structures (Fig. 3c). This model is based on surface-level destabilization and complete membrane disintegration, making it different from the aggregate model, which is also based on the formation of peptide-lipid aggregates or micelle-like complexes. In the aggregate model, although the AMPs do not form pores in the traditional sense, the peptides may penetrate the membrane creating transient channels or micellar structures that allow ion leakage and intracellular entry of peptides, enabling both membrane disruption and intracellular targeting (Fig. 3d) [104].

In humans, AMPs are produced by 1) innate immune cells and less frequently by 2) adaptive immune cells and 3) barrier forming cells, such as intestinal epithelial cells (Paneth cells and mucosal membrane cells). The main human AMPs are defensins, histatins, cathelicidin, dermcidin and hepcidin. While defensins, cathelicidin and dermcidin attack by disrupting microbial membranes, histatins and hepcidin act through other mechanisms, such as ion chelation, signaling, or enzyme inhibition. Below we discuss the mechanism of action of the main pore-forming AMPs.

##### Defensins

Human defensins are composed of around 30–40 amino acids and have a molecular weight of 3–5 kDa [108, 109]. They are produced as pre-pro-peptides and undergo processing either within the cell during synthesis or outside the cell, after secretion, depending on the specific type of defensin and the cell in which it is expressed. For example, neutrophil α-defensins are synthesized in promyelocytes as a 94-amino acid-long pre-pro-defensin, which undergoes proteolytic processing. First, the N terminal signal sequence is removed producing an approximately 75-amino acid long pro-defensin [110]. Further proteolytic processing of the N-terminus reduces the length to 29–30 amino acids, producing the mature defensin. Mature human defensin molecules contain six cysteine residues that form three intramolecular disulfide bonds, stabilizing a three-stranded antiparallel β-sheet [110].

Human defensins (HD) are classified into α- and β-forms based on the spacing between the six cysteine residues and the pattern of the disulfide bonds. The alpha defensin family have six members (human neutrophil defensin (HNP) 1–4, HD5 and HD6), while the β-defensin group has at least 8 members [111] with current genomic approaches suggesting the existence of up to 28 more β-defensin genes [112–114], but some of these are pseudogenes, not producing a defensin protein.

The synthesis and release of defensins are regulated in a tissue-specific manner by microbial signals, developmental cues, cytokines and neuroendocrine signals. Human neutrophil defensins are produced constitutively by bone marrow precursor cells during specific stages of neutrophil differentiation, particularly in promyelocyte and early myelocyte stages. After synthesis, these defensins are stored in primary azurophil granules. During phagocytosis of microbes, the azurophil granules merge with the phagocytic vesicles, delivering high concentrations of defensins to kill the pathogens [110, 115].

Defensins are positively charged, which enables them to bind to negatively charged bacterial membrane components, such as lipopolysaccharides (LPS) in Gram-negative bacteria, polysaccharides and teichoic acids in Gram-positive bacteria and phosphatidylglycerol in the membranes of both Gram-negative and Gram-positive bacteria [116, 117]. When defensins bind to the membrane, especially LPS moieties, they competitively displace the Ca^2+^ and Mg^2+^ ions which bridge the LPS molecules to stabilize the LPS layer [118]. Due to displacement of membrane-stabilizing ions, and much larger than LPS monomers, defensins thin the membrane, thus weakening its barrier function and enhancing its permeability. Defensins can also directly perforate the bacterial membrane [119]. Although the exact mechanism of it is not well understood and at present there are a number of proposed mechanisms how defensins cause membrane rupture. For example, the Rhesus macaque myeloid alpha defensin-4 (RMAD4) has been shown to perforate membranes via the carpet model [120], while human neutrophil peptide-1 (HNP-1) is likely to form barrel-stave pores [22]. The actual mechanism of membrane perforation is under-investigated and it is likely to be complex due to the versatility of defensins as well as the heterogeneity of the microbial membranes they may target [121]. Regardless of the process of membrane permeabilization, electrolyte leakage and membrane depolarization takes place, inhibiting DNA, RNA and protein synthesis, as well as respiration, culminating in cell death [116, 122].

##### Cathelicidin

Cathelicidins, first identified in myeloid cells, are named so due to the presence of a conserved cathelin domain in their N-terminal pro-region, which has high similarity to the cathepsin L inhibitor of the same name. Over 30 members of the cathelicidin family have been identified so far in mammalian species, with a single cathelicidin expressed in humans (hCAP18) [123, 124]. The structure of cathelicidins consists of an N-terminal signal peptide, followed by the conserved pro-region (cathelin domain) of about 100 residues containing two disulfide bonds. The C-terminal segment contains the antimicrobial peptide domain, which exhibits considerable diversity across species, indicating the evolutionary pressure to combat pathogens, forcing the gene to evolve with the pathogens, Accordingly, the C terminal domain can have both α-helical or β-hairpin folds, as well as proline and arginine rich structures [125, 126]. Regardless of the varied fold, the C-terminal domain is cationic and amphipathic to facilitate binding to microbial membranes [127].

Human cathelicidin is stored in an inactive state (also called: hCAP-18; where the N-terminal pro-region is connected to the C-terminal AMP) in granules of neutrophil granulocytes and released in this form upon neutrophil activation [128]. Once released, the also secreted neutrophil elastase cleaves off the pro-region thus releasing the mature AMP [119, 129, 130]. The formed, biologically active peptide was named as ‘LL-37’ when it was first isolated from neutrophils and identified as a 37-amino-acid sequence starting with two leucine residues [131]. Currently, “hCAP18” name refers to the precursor cathelicidin, while LL-37 designates the mature peptide [132]. A single gene on chromosome 3, (3p21.3) codes for hCAP18/LL-37 and is primarily expressed in the bone marrow, mucosal epithelial cells and keratinocytes of inflamed sites [133].

In epithelial tissues, LL-37 exhibits moderate antimicrobial activity against a wide range of Gram-negative and Gram-positive bacteria, including species from the *Pseudomonas*, *Escherichia*, *Staphylococcus*, and *Enterococcus* genera [134]. Its primary antibacterial action is disrupting bacterial membranes. The peptide’s + 6 net positive charge enables it to stably bind to the negatively charged bacterial membrane, leading to the formation of transmembrane pores, although the mechanism is debated, with some evidence pointing towards the carpet model [135, 136] as well as the barrel-stave model [137, 138]. Similar to other PFPs, membrane permeabilization compromises cell integrity, resulting in lysis and death [139]. Additionally, inside microbes, LL-37 can also target intracellular components, such as acyl carrier proteins important for lipid synthesis and cell membrane biogenesis, also contributing to its cytotoxicity [139, 140].

## Intracellular pore-forming proteins of the immune system

Membrane integrity is central to a cell’s survival. They enable compartmentalization of processes, trafficking of molecules and establishment of a membrane potential. Sustained disruption of membrane integrity is thus incompatible with life and marks the point of no return in cell death processes [141]. Loss of membrane integrity is the defining event of necrotic cell death, when cells burst open due to extensive deregulation of ion homeostasis. However, loss of membrane integrity is also an integral part of RCD pathways, including apoptosis, pyroptosis, ferroptosis, NETosis as well as necroptosis and can take place in both intracellular membranes and the plasma membrane [142].

The best known and classical examples of intracellular pore-forming proteins are the pro-apoptotic Bcl-2 family proteins, Bax and Bak, known for their function of forming large, toroid pores in the mitochondrial outer membrane [143, 144]. Perforation of the outer mitochondrial membrane dissipates the mitochondrial membrane potential required for mitochondrial ATP production. Additionally, with the loss of outer mitochondrial membrane integrity, mitochondrial intermembrane space proteins get released into the cytosol, where many of them act as pro-apoptotic factors. These proteins include cytochrome *c* (Cyt *c*), which activates Apaf-1 thus initiating the assembly of the Apaf-1-based apoptosome complex activating pro-caspase-9, SMAC/DIABLO, which relieves caspase-9 and -3 inhibition exerted by X-linked inhibitor of apoptosis protein (XIAP), or AIF (apoptosis-inducing factor), which triggers large-scale DNA fragmentation, processes that collectively commit the cell to death [145].

During the past decade it emerged that many RCD pathways culminate in the activation of intracellular PFPs. For example, necroptosis signaling leads to activation and pore formation by MLKL, while pyroptosis and NETosis concludes in the activation and membrane pore formation by gasdermins and NINJ1. The section below discusses the intracellular PFPs that function in mammalian organisms.

### The antibacterial perforin: Perforin-2

Perforin-2, coded by the MPEG1 gene, is an immune effector protein that exhibits dual antimicrobial function: it perforates bacterial membranes to lyse bacteria and it also activates proinflammatory signaling, thereby maximizing effectiveness [146]. Similar to PRF1, PRF2 belongs to the MACPF/CDC protein superfamily; however, they differ in tissue specificity, targets, subcellular localization and mechanisms of action. While PRF1 is primarily expressed by cytotoxic lymphocytes and functions extracellularly to eliminate infected or malignant cells, PRF2 is constitutively expressed in cells of monocytic origin, such as macrophages, and contributes to the elimination of intracellular bacteria, including Gram-positive, Gram-negative and acid-fast bacteria [147]. Its expression can also be induced in barrier forming cells, such as epithelial cells and fibroblasts by interferons (INFα, β, and γ) upon bacterial infection [148].

Unlike PRF1, PRF2 is a transmembrane protein and consists of a short signal peptide (SP), an ectodomain, and an endodomain. The SP directs PRF2 to the ER after which it is proteolytically removed. Within the ER membrane, the ectodomain is strategically oriented towards the luminal space, while the endodomain is exposed to the cytosolic face. The ectodomain contains the MACPF domain with two sets of TMH1 and TMH2 motifs, followed by an EGF domain, a multi-vesicular body-12 (MVB12)-associated b-prism (MABP) domain (also known as P2 domain or β-hairpin) and a C-terminal tail (CTT) or L-domain. The endodomain includes a single-pass transmembrane domain (TMD) followed by a short cytosolic sequence [24, 149].

Upon bacterial infection, PRF2 is transported to phagosomes to kill endocytosed bacteria. PRF2 may also localize to the cell surface where it facilitates the elimination of extracellular pathogens [10, 146]. It has to be noted that the activity of PRF2 is very different depending on its localization. On the cell surface, it appears to interact with and facilitate signaling through type I interferon receptors (IFNAR1 and IFNAR2) [10], while its pore-forming activity is active intracellularly, restricted to the phagosome.

**Fig. 4:**
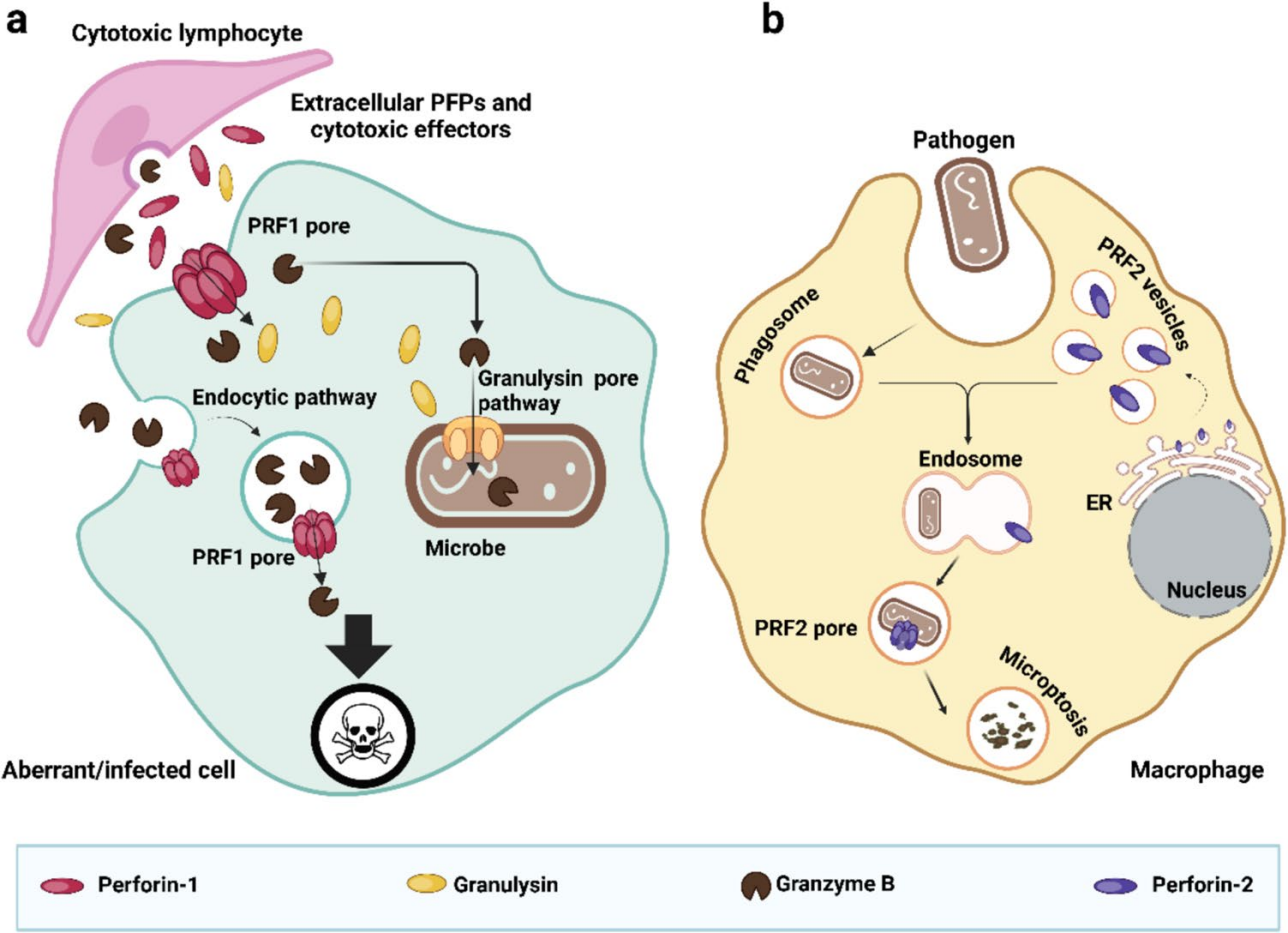
Intracellular and extracellular pore-forming activity of perforins. (a) Perforin-1 (PRF1) is exocytosed by cytotoxic lymphocytes into the synaptic cleft along with cytotoxic molecules. PRF1 then creates pores in the plasma membrane of the targeted, abnormal (infected, damaged or aberrant) host cell causing cell lysis and delivery of other cytotoxic proteins, such as granzymes (GZMs) and granulysin directly through PRF1 pores or indirectly, via endocytosis of PRF1-perforated membrane segments with GZMs attached to it. (b) In contrast, perforin-2 (PRF2) primarily acts intracellularly. PRF2 is stored in the endoplasmic reticulum (ER) as a transmembrane protein and transported to phagosomes where it attacks engulfed microbes by perforating their membrane. Figure was generated with BioRender

Microbe phagocytosis, LPS and IFN-gamma signaling induces monoubiquitylation of conserved lysine-rich motifs in the cytosolic tail of PRF2 by the cullin ring ligase (CRL) and β-Transducin Repeat Containing Protein (βTrCP) complex, which triggers the transport of PRF2 to endosomal/phagosomal bodies. Once in the phagosome membrane, PRF2 assembles into ring-shaped pre-pores of ~ 12–13 nm, with the N terminal, MACPF domain being in the phagosome lumen. The pre-pore complexes may be detached from the phagosome membrane either by phagosome acidification or proteolytic cleavage. Once released, the pre-pore complexes bind to negatively charged lipids present in microbial membranes and the acidic pH induces the assembly of the pre-pores into mature pores to lyse bacteria [150]. It is also possible, that the PRF2 pre-pore complexes remain tethered to the phagosome membrane via the MABP domains while the MACPF domains form a pore in the bacterial membrane [151]. Compared to PRF1 pores, which facilitate the delivery of cytotoxic effectors to target cells and initiate regulated cell death, PRF2 pores operate through a simpler mechanism of osmotic lysis (Fig. 4).

There are several mechanisms that preserve the host membrane form PRF2-induced damage. The neutral pH at the ER and Golgi act as a regulatory mechanism preventing activation until PRF2 reaches the phagosomes [151]. Within the phagosomes, the TMD and the MABP domains serve as a crucial structural element anchoring PRF2 to the membrane oriented in a way that the pore-forming MACPF domains face away from the phagosome membrane, thereby preserving the host membranes from PRF2 pore activity. Finally, PRF2 pores are preferably formed on negatively charged microbial membranes [24].

### Gasdermins

Pyroptosis is a form of immunostimulatory RCD that typically occurs in macrophages upon pathogen infection. It is however not limited to immune cells; epithelial cells, endothelial cells and some other cell types can also undergo so-called sterile pyroptosis (pyroptosis induced by cellular stress and damage as opposed to pathogens and mediated by DAMPs), e.g. during mucosal barrier dysfunction in Crohn’s disease [152] or in response to certain anticancer drugs [153]. Pyroptosis can be executed through four main pathways including inflammasome-dependent (canonical and non-canonical pathways) and independent mechanisms (caspase-3 and GZM-mediated pathways) [154]. Pyroptotic cells are characterized by osmotic imbalance and membrane rupture, leading to ion exchange and rapid release of pro-inflammatory mediators and alarmins (such as interleukin (IL)-18, IL-1β, high mobility group box 1 protein (HMGB1) and others) into the extracellular space and activating an inflammatory response.

**Fig. 5:**
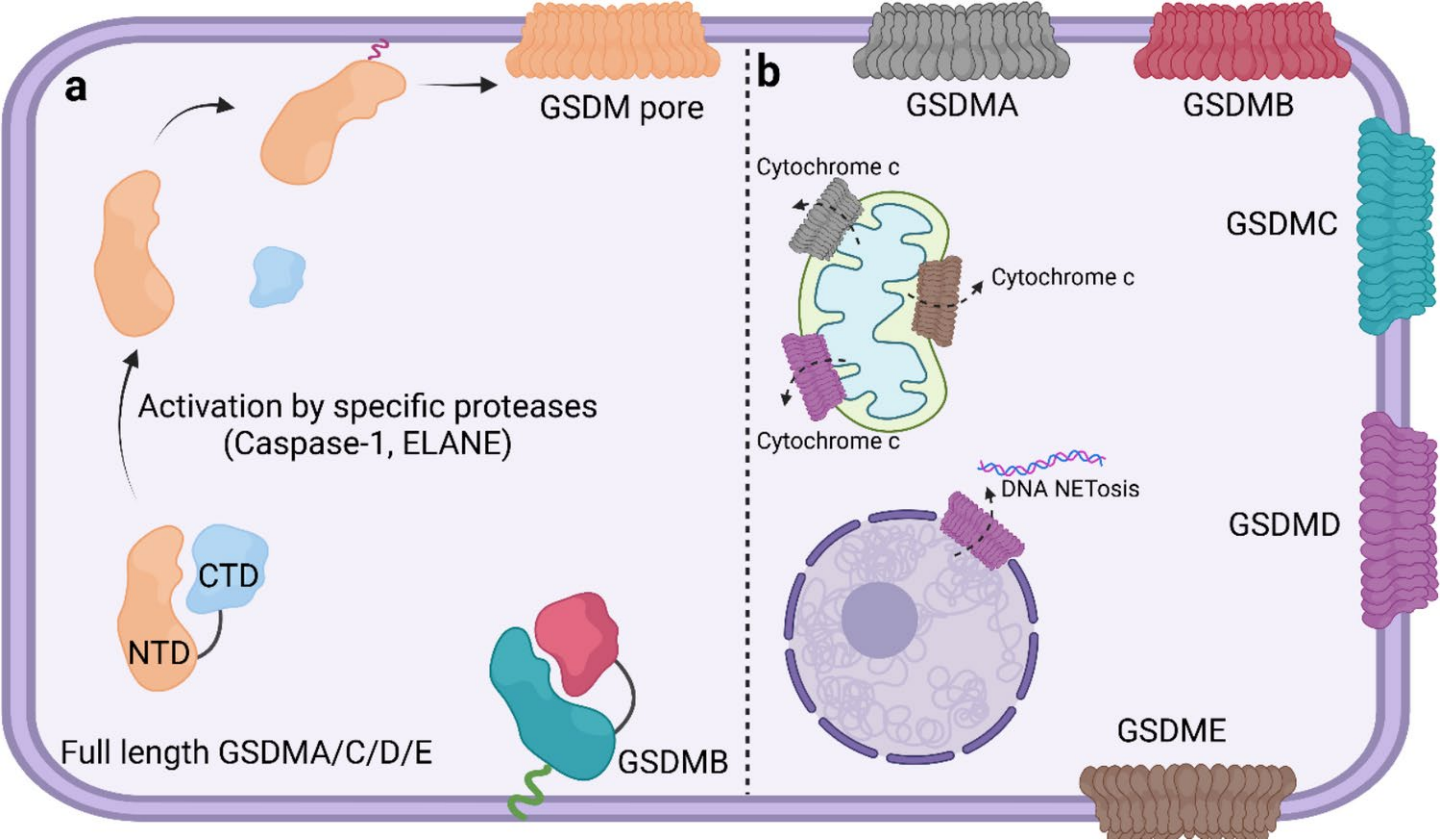
Schematic illustration of the activation and mechanism of function of gasdermin proteins. (a) Full-length gasdermins (GSDMs) are intercellular proteins primarily localized in the cytosol, except for GSDMB, which binds to the membrane. When the inhibitory C-terminal domain is removed by proteolytic cleavage, releasing the N-terminal domain, which leads to plasma membrane rupture (PMR). (b) GSDMs predominantly target the plasma membrane, while some, including GSDMA, GSDMD, and GSDME, also target intracellular organelles. Figure was generated with BioRender

GSDMs, a family of homologous PFPs, are regarded as the central executioners of pyroptosis with six known members in humans: GSDMA, GSDMB, GSDMC, GSDMD, GSDME, and PJVK [155]. All GSDMs, except PJVK, share a conserved domain architecture consisting of an amino-terminal pore-forming domain (PFD) and a carboxy-terminal repressor domain (RD). The N-terminal domain consists of three α-helices and ten β-strands, while the C-terminal, autoinhibitory domain contains nine α-helices capped by a three-stranded β-sheet [156]. A flexible and member-specific linker connects the two domains, ensuring that the full-length GSDM remains in an auto-inhibited conformation. In this state, the C-terminal repressor domain masks the N-terminal pore-forming fragment, locking the protein in a globular, inactive state [157]. GSDMs are classified as intracellular PFPs and are predominantly localized in the cytoplasm in their autoinhibited state [158]. The exception is GSDMB, which exhibits a unique ability to directly bind to the inner surface of the plasma membrane due to the absence of two α-helices in its C-terminal fragment (Fig. 5a) [159].

In response to various exogenous pathogens and endogenous damage, GSDMs are proteolytically cleaved at the linker region by specific proteases, such as caspases, neutrophil elastase (ELANE), Streptococcal pyrogenic exotoxin B (SpeB), or GZMA/B. For instance, microbial infections and internal danger signals induce the assembly of the canonical inflammasome complexes which activate pro-caspase-1. Activated caspase-1 then cleaves GSDMD at its middle linker, releasing the active, N-terminal fragment, which then permeabilizes the plasma membrane from the inside. GSDMD pores provide a route for the rapid release of alarmins/or DAMPs, such as lactate dehydrogenase (LDH), IL-18, IL-1β, DNA-binding histones, and HMGB1, which initiate inflammatory responses [160, 161]. The pores can also cause permanent plasma membrane rupture (PMR), leading to pyroptosis-induced cell death [162]. In a similar proteolytic mechanism, caspase-4/5 and caspase-3 have also been shown to activate GSDMD and GSDME, respectively during bacterial infections [163].

In addition to caspases, GSDMD can also be cleaved by ELANE, a neutrophil-specific serine protease present in activated neutrophil granulocytes. Similar to macrophages, inflammasome activation in neutrophils leads to pro-caspase-1 activation and caspase-1-mediated processing of GSDMD. The resulting free amino-terminal subunit of GSDMD however, instead of the plasma membrane, targets the azurophilic granules of the neutrophil granulocytes to release the granule’s content into the cytosol. The released serine proteases, including ELANE then cleave other GSDMD molecules, leading to a feed-forward cascade of GSDMD processing finally culminating in plasma membrane permeabilization and NETosis, a neutrophil-specific cell death mechanism characterized by the release of DNA complexed with antimicrobial enzymes and AMPs (neutrophil extracellular traps, NETs) [164–166].

SpeB is protease that cleaves GSDMA, which is predominantly expressed in the skin during *Streptococcus pyogenes* infection. The cleavage liberates the PFD of GSDMA which then forms pores causing keratinocyte pyroptosis [167, 168]. GZMA/B are the last group of proteases that contribute to the activation of GSDMB/E.

Upon release from the autoinhibitory interaction with the C-terminal domain, positively charged and amphipathic regions of the NTD get exposed and interact with negatively charged lipids, such as cardiolipin, phosphatidylinositol phosphates (PIPs) and phosphatidylserine (PS), present in mitochondrial membranes and in the inner leaflet of the plasma membrane of mammalian cells [17, 169]. Of note, cholesterol inhibits the initial stage of GSDMD binding to the lipid membrane. However, once GSDMD binds and inserts into the membrane, cholesterol does not interfere with the self-assembly of oligomeric transmembrane pores [170].

After the free NTD inserts into the membrane, 26–34 NTD units oligomerize into a 20 nm diameter β-barrel pre-pore intermediate held together by lateral interactions between GSDM monomers. In these pre-pores, the N-terminal globular ends of the NTD units (heads) remain largely unchanged, while in the pore-forming segments undergo a radical conformational change, where they reorganize into two anti-parallel β-hairpins (fingers), long enough to transverse the lipid bilayer. The transmembrane β-hairpins are amphipathic, with the side facing the lipid bilayer being hydrophobic while the side facing the lumen of the pre-pore contains positively and negatively charged patches. The pre-pores are partially immersed in the membrane and the globular heads of the GSDM units stay above the membrane, are tilted, bending towards the β-hairpins. The transition from the pre-pore stage to the mature pore is facilitated by an approximately 38° rotation of the globular heads upward, away from the membrane and the β-hairpins. This rotation occurs during full insertion of the β-strands into the membrane, changing the shape of the protein to allow inter-monomer interactions and eliminate steric hindrances [171].

The current model of GSDM pore formation described by Mulvihill and colleagues is somewhat different from the above model for human GSDMD pores. Time-lapse experiments demonstrated that the heights of the arc-, slit- and ring-shaped oligomers did not show a vertical collapse during pore formation, suggesting the absence of a pre-pore state [170].

While the mechanism of pore-formation is comparable across family members, it is not true for the lipid binding affinities for GSDMs. This difference between individual GSDMs may explain the differential kinetics of GSDM proteins in binding to different membranes. For instance, the N-terminal fragment of GSDMA shows a strong preference for binding to cardiolipin and a weaker preference for PIPs, compared to GSDMD. Consequently, GSDMA exhibits a time-dependent subcellular localization pattern, with an initial localization to the mitochondria and a delayed redistribution or localization to the plasma membrane. In contrast, GSDMD rapidly targets both mitochondria and the plasma membrane [172]. In another scenario, binding of GSDMD to mitochondria leads to activation of pro-caspase-3, which subsequently cleaves GSDME, suggesting a potential crosstalk between GSDMD and GSDME in regulating cell death pathways [173, 174]. Moreover, the presence of cardiolipin in the mitochondrial membrane serves as a primary target for the N-terminal fragment of GSDME, which propagates cytochrome *c* release, initiating apoptosome formation and effector caspase activation. This, in turn, creates a self-amplifying loop that promotes further cleavage of GSDME, ultimately resulting in PMR (Fig. 5b) [175].

Generally, GSDM pores result in two distinct outcomes in mammalian cells, which are cell death on one hand, characterized by PMR and pyroptosis, and non-lethal functions on the other hand, that facilitate the rapid release of inflammatory factors [154]. Induction of cell death requires membrane rupture, which may be achieved by some of the above-described feed-forward loops amplifying GSDM activation, or via activation of additional PFPs, such as NINJ1 (see Sect. ”Ninjurin-1”).

To ensure that GSDM do not trigger unwanted cell death, mammalian cells employ two interacting mechanisms to restore membrane integrity: activation of the endosomal sorting complex required for transport (ESCRT) system and the membrane repair system. The ESCRT system is a conserved membrane remodeling machinery that regulates the scission and repair of cellular membranes. For example, upon GSDMD pore formation, the influx of Ca^2^⁺ ions activate the ESCRT-III complex initiating its recruitment to the cell membrane. Here the complex pinches off the pore-containing, leaky membrane sections and sheds them as ectosomes [176]. In another scenario, excessive influx of Ca^2^⁺ through GSDM pores leads to lysosome exocytosis, releasing acid sphingomyelinase (ASM) onto the cell surface where it converts sphingomyelin into ceramide. Caspase-7, activated by caspase-1 during pyroptosis can also cleave and activate ASM, thereby increasing ceramide levels. Ceramide induces membrane endocytosis by enforcing negative membrane curvature and invagination, thus removing the membrane segments that contain GSDMD pores, thus restoring membrane integrity and preventing pyroptotic cell death [177]. In addition to the intracellular mechanisms controlling and restricting GSDM-mediated membrane perforation, it has to be noted that the selectivity of GSDMs for negatively charged lipids and phospholipids restricted to the inner leaflet of mammalian cell membranes serves as a protective mechanism to protect bystander cells from GSDM perforating activity.

### Ninjurin-1

The nerve injury-induced protein, or ninjurin protein family consists of two members in humans: NINJ1 and NINJ2. Of the two proteins, only NINJ1 has been identified as a membrane pore-forming protein. Originally discovered as a protein induced upon nerve injury, NINJ1 has since emerged as a multifunctional protein. NINJ1 is highly expressed in neuronal and Schwann cells, where it facilitates axonal growth and regeneration after peripheral nerve damage [178–180]. Beside the nervous system, NINJ1 is also found in various tissues and cell types, including endothelial cells [179] where it plays a role in vascular homeostasis and microvessel maturation following vascular injury. In many other cell types, NINJ1 acts as an executioner of programmed cell death pathways, especially pyroptosis and ferroptosis by causing PMR [11, 181].

Unlike other pore-forming proteins, NINJ1 constitutively localizes to the plasma membrane. Structurally, it consist of 4 alpha helices and an unstructured N terminal section, with helices-1 and -2 forming an N-terminal extracellular domain and α3 and α4 spanning the membrane [181]. Helices-3 and -4 are oriented in a parallel manner, where one face of the transmembrane segment is hydrophobic, and the other is hydrophilic. In its resting state, NINJ1 exists in an autoinhibited configuration as a dimer or tetramer, where the hydrophilic faces of two NINJ1 molecules are aligned against each other, preventing activation and membrane rupture [181].

The best understood mechanism of NINJ1 activation is that of during pyroptosis where NINJ1 acts downstream of GSDMD [181, 182]. GSDM pore formation during pyroptosis triggers so-far not understood changes that activate NINJ1, upon which NINJ1 undergoes a conformational shift. The extracellular helices α1 and α2 insert into the membrane where α2 aligns parallel with α3 and α4, while α1 reorients to be nearly perpendicular to the other helices [26, 181, 183]. The angled α1 acts as an arm to link to an adjacent NINJ1 monomer, forming long, fence-like, filamentous structures of up to 2 µm length where the hydrophilic faces of the NINJ1 units are exposed. These filaments can cap membrane edges by binding to the lipids via the hydrophobic face of the filament and disrupting membrane continuity via the hydrophilic face. The membrane capping generates several, large pores of variable sizes (more than 10–50 nm), ultimately leading to PMR and cell lysis [26].

PMR facilitates the release of intracellular DAMPs such as HMGB1 [184]. This amplifies the inflammatory response in the tissue by recruiting immune cells and enhancing cytokine signaling. The loss of membrane integrity ultimately results in complete cell lysis, marking the terminal phase of pyroptosis and other forms of cell death.

NINJ1’s role extends beyond pyroptosis, as it has been implicated in other forms of cell death, including ferroptosis, necroptosis and apoptosis, although its contribution to these death programs is less understood [184]. Regulation of NINJ1 is another area of active research and so far, poorly understood. It has been suggested that matrix metalloproteinases (MMPs) may interact with NINJ1, potentially regulating its activity [185]. Furthermore, the amino acid, glycine has been shown to inhibit NINJ1-mediated pore formation, suggesting that molecular interactions may modulate NINJ1’s function [186]. Understanding these regulatory mechanisms will be essential to elucidate how NINJ1 contributes to cell death execution and inflammation.

### Mixed lineage kinase domain like pseudokinase

MLKL is expressed across a wide range of vertebrates, including mammals, birds, reptiles, and fish, highlighting its evolutionary significance. MLKL functions as the terminal executioner of necroptosis, a regulated form of cell death, which despite its controlled nature, exhibits morphological similarities to the accidental cell death of necrosis [187]. Necroptosis has likely evolved as an alternative or fail-safe cell death program for pathogen-infected cells in which the core apoptotic pathway is compromised, for example, by the caspase-8 inhibitor, vFLIP (viral FLICE-inhibitory protein), released by viruses [188]

Necroptosis can be induced by various stimuli, including death ligand cytokines, lipopolysaccharides and double-stranded DNA detected by Toll-like receptors, inflammatory cytokines activating their receptors (interferons, TNF), and cytosolic Z-DNA and Z-RNA (left-handed helical nucleic acids) detected by the cytosolic nucleic acid sensor, ZBP-1 (Z-DNA Binding Protein-1). Signaling from these diverse receptors converges on the activation of the serine/threonine kinase RIPK3 (receptor-interacting protein kinase-3). The prototypical necroptosis pathway, where the steps of the signal transduction pathway leading to RIPK3 activation and necroptotic cell death are best understood is the one initiated by the death ligand, TNF. When TNF binds to its death receptor, TNF-R1, the receptor triggers three main signaling pathways[189].

The first pathway activates the transcription factor, nuclear factor kappa B (NF- ΚB). Upon activation, the membrane-bound TNF-R1 receptor recruits the adaptor proteins, receptor-interacting protein kinase-1 (RIPK1), TNF-R1-associated death domain protein (TRADD) and TNF receptor-associated factor-2 (TRAF2) to which the ubiquitin ligases cIAP1 and -2 (cellular inhibitor of apoptosis proteins 1 and 2) and linear ubiquitin chain assembly complex (LUBAC) proteins bind, forming a high molecular weight protein complex, termed as Complex I. In the complex, cIAP1/2 and LUBAC polyubiquitylate RIPK1, TRADD, and TRAF2. NF-ΚB activating adaptor and kinase complexes (TAB1/2, TAK1, NEMO, and IKK1/2) then dock onto these polyubiquitin chains and induce NF-ΚB activation.

**Fig. 6:**
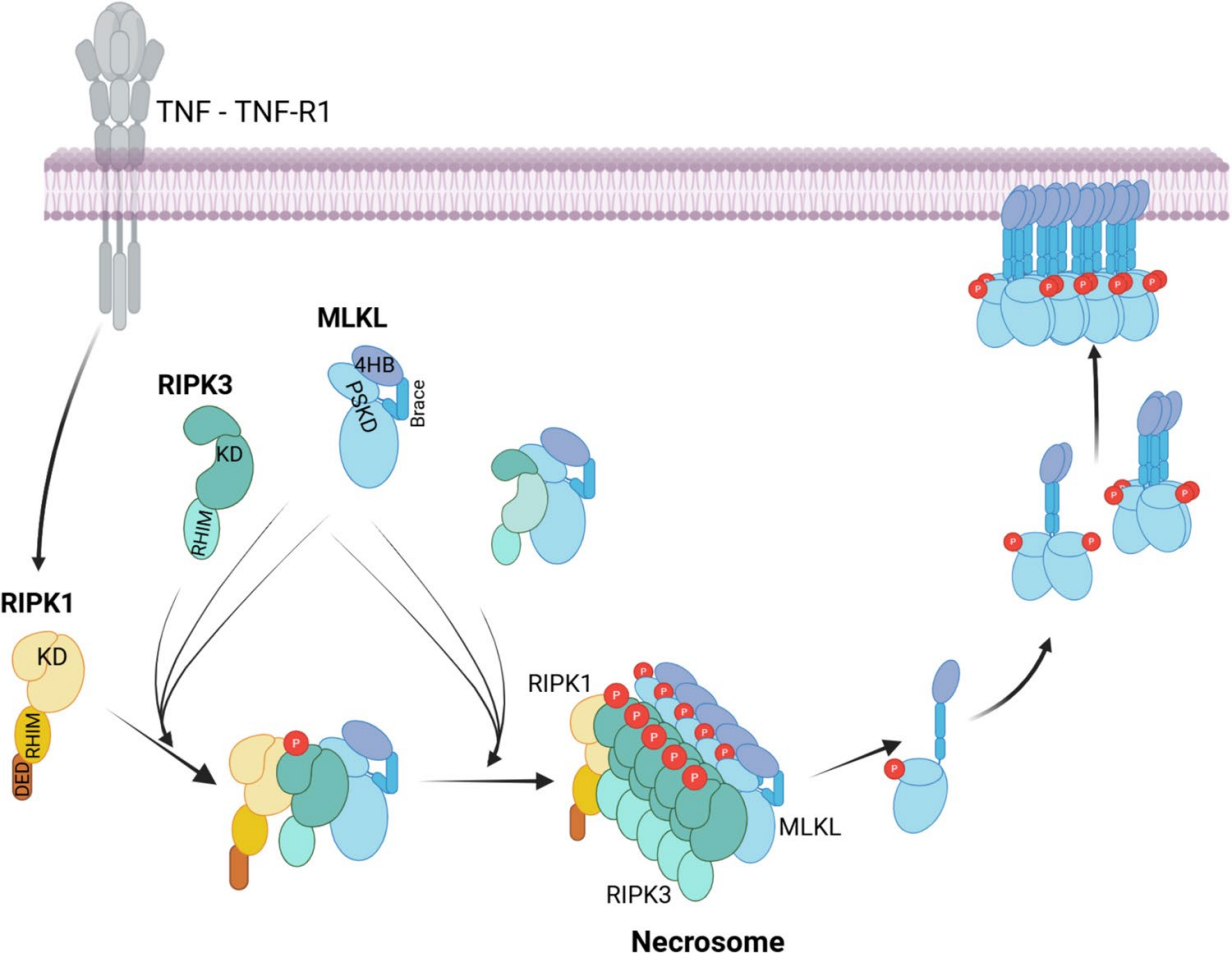
Mechanism of mixed lineage kinase like pseudokinase membrane permeabilization during necroptosis. Activation of tumor necrosis factor receptor-1 (TNF-R1) by TNF initiates necroptotic signalling when the adaptor protein, receptor interacting protein kinase-1 (RIPK1) binds to the receptor, but does not get polyubiquitylated. This may happen if cellular apoptosis inhibitor proteins (cIAP)1/2 and/or the LUBAC ubiquitylating protein complexes do not bind to TNF-R1 (not shown), or de-ubiquitylating enzymes (e.g. A20) are recruited (not shown). Non-ubiquitylated RIPK1 is then released into the cytosol and binds several RIPK3 and MLKL units either as monomers or as inactive heerodimers. RIPK1 phosphorylates and thus activates RIPK3 in the complex, changing its kinase domain (KD) from its open, inactive conformation to the closed, active conformation. RIPK3 then phosphorylates MLKL in its pseudokinase domain (PSKD). Phosphorylated MLKL detaches from the necrosome and undergoes a structural transition. Its PSKD switches to the closed, active-like conformation that triggers the release of the brace region, which rearranges into an elongated helix. The elongated brace helices of 2 MLKL dimers then combine into a tetrameric coiled coil. Finally, the 4HB domains twist away from the brace region, undergo a conformational change that exposes a positively charged pocket and an amphipathic organization for sinking into the lipid bilayer. These MLKL units are trafficked to the cell membrane where they lead to membrane permeabilization. Figure was generated with BioRender

The second signaling pathway initiated by TNF-R1 leads to apoptosis. This occurs when ubiquitylation fails to take place in Complex I, for example due to the absence or inhibition of cIAP1/2. In the absence of ubiquitylation, the adaptor proteins detach from the receptor. As they detach, TRADD and RIPK1 nucleate an alternative signaling complex in the cytosol, termed Complex II, by recruiting another adaptor protein, Fas-associated via death domain protein (FADD), which in turn recruits pro-caspase-8. Pro-caspase-8 becomes activated in Complex II via induced proximity (dimerization and/or polymerization), and initiates the apoptosis signaling cascade (Fig. 6).

The third, signaling pathway is necroptosis. It is activated when pro-caspase-8 activation is blocked in Complex II, for example by the viral caspase-8 inhibitor, vFLIP. In this scenario, RIPK1 in Complex II recruits another kinase, RIPK3, and the PFP, MLKL, forming a new protein complex, called the necrosome. RIPK3 oligomerizes in the necrosome and becomes autoactivated through cross-phosphorylation by adjacent RIPK3 units. MLKL is then phosphorylated by RIPK3 on threonine 357 and serine 358 (T357, S358) in its pseudokinase domain [190, 191]. This phosphorylation induces a conformational change in MLKL facilitating its translocation to the inner surface of the plasma membrane where it binds to negatively charged phospholipids, such as PS and PIPs [192]. Upon membrane binding, MLKL oligomerizes to form higher-order structures and submerges into the lipid bilayer, creating pores. These pores, characterized as small (1 – 4 nm diameter), cation-selective channels, permit the passage of ions and small molecules through the membrane, leading to osmotic imbalance, cell swelling, and ultimately, cell death [27, 193, 194].

The importance of the necroptotic cell death pathway is underscored by the fact that several pathogenic microbes produce necroptosis inhibitors (e.g. MLKL inhibitors) to evade host cell death, thereby enhancing their virulence [188]. As a counter measure, the MLKL gene has undergone significant evolutionary divergence. Studies reveal that while the overall structure of MLKL is conserved, its amino acid sequence and regulatory mechanisms vary greatly among species [195, 196]. For example, the amino acid identity between human and rodent MLKL is only 62%, making MLKL even from closely related species to be non-interchangeable [195, 197]. Consequently, in the sections below, the review focuses on characteristics of human MLKL, unless stated otherwise.

The conserved structural units of MLKL include three regions. The N-terminus contains a unique coiled coil, four-helix bundle (4HB) or HeLo domain which mediates lipid binding, followed by an intermediary two-helix “brace” region that facilitates MLKL oligomerization. Finally, the C-terminal segment contains a pseudokinase domain, which is essential for the regulation of MLKL activity [198–201].

The activity of MLKL is controlled by phosphorylation. Unphosphorylated MLKL is inactive and present in the cytosol either as a monomer [202] or in a heterodimeric form with RIPK3 [203]. RIPK3-MLKL dimers are formed when RIPK3 is phosphorylated on serine-227 (S227) by itself or by casein kinase-1 family kinases, as phosphorylation of this site is essential for the stable binding of MLKL [194, 204]. The inactive conformation of MLKL in both the monomeric and RIPK3-dimeric forms is maintained by interactions of the 4HB domain with the brace helices and the pseudokinase domain, which hold the 4HB domain in a restrained conformation (Fig. 6). Of note, although RIPK3 is the activator of MLKL, in the RIPK3-MLKL heterodimer the activating phosphorylation sites of MLKL are far from the catalytic pocket of RIPK3. Furthermore, RIPK3 adopts an inactive, open conformation in the dimer, corroborating the notion that the non-necrosome associated dimer is inactive [203].

During necroptosis signaling RIPK1 recruits RIPK3 units (possibly both monomers and RIPK3-MLKL dimers) to the necrosome. The joining RIPK3 units get aligned through their N-terminal RHIM (RIP homotypic interaction motif) domains. In this structure, RIPK3 can be phosphorylated on S227 by CK1 kinases also present in the necrosome, and on S232 and S369 by RIPK1. These phosphorylation events (S369, S232) facilitate the stable binding of RIPK3 to the necrosome. Additionally, adjacent RIPK3 units can cross-phosphorylate each other on residues T224 and S227, which triggers the recruitment and consequent phosphorylation of MLKL on T357 and S358 (or a subset of the T355, T357, S358 and S360 residues, [205]) in its pseudokinase domain.

The structure of the phosphorylated, active human MLKL is incompletely understood. The current best model, supported by crystallographic and negative-stain electron microscopy, suggests that RIPK3-mediated phosphorylation pushes the MLKL pseudokinase domain to switch into a closed, active-like conformation, which in turn drives MLKL dimerization [205]. The conformational change in the pseudokinase domain also releases the brace region, which then rearranges into an elongated helix. The elongated brace helices of 2 MLKL dimers then combine into a tetrameric coiled coil, with the pseudokinase domains sitting at the bottom of the coiled coil and the 4HB domains on the top. The 4HB domains get distanced from the brace helices (plug-release mechanism) [199] and assemble into an α-helical bundle [205]. Assembly of the tetrameric 4HB bundle also exposes a positively charged pocket, a site likely to interact with negatively charged membrane lipids and capable of membrane permeabilization [206]. The MLKL tetramers or oligomers are trafficked to the membrane via a Golgi-microtubule-actin-dependent mechanism, rather than via random diffusion were they gradually accumulate into μm-sized membrane hotspots [207].

The supramolecular structure of the MLKL oligomers at the membrane remains undefined, too [142]. Early studies proposed that rather than directly permeabilizing the membrane, MLKL causes cell lysis by activating an endogenous ion channel (the transient receptor potential melastatin-related 7, TRPM7 channel) [208, 209], or MLKL itself forms an ion channel [27]. However, recent data showed that this model is unlikely, as the proposed MLKL-targeted ion channels were not essential for necroptosis [27, 209, 210].

A more general hypothesis is that MLKL directly mediates the permeabilization of the plasma membrane either as a result of its partial insertion into the lipid bilayer [199] or by forming membrane-spanning channels or pores [27] leading to osmotic swelling and PMR. MLKL trafficking and accumulation into variable, micrometer-range hotspots at the plasma membrane act as crucial checkpoints for necroptosis [207]. In this model, the binding of MLKL oligomers to the membrane might induce flipping of the hydrophobic core of the MLKL 4HB domain to create an amphipathic organization of hydrophobic and hydrophilic faces that enables the assembly of a toroidal pore [142]. Alternatively, the amphipathic α-helices of the 4HB domain may form a carpet surfactant and thus drive membrane rupture [199, 211].

Similarly to GSDMs, MLKL cytotoxicity is counterbalanced via the membrane repair mechanisms mediated by the ESCRT-III secretory system and the endocytic membrane repair machinery [212], suggesting that MLKL-mediated cytotoxicity might also require inhibition of these membrane repair pathways.

## Therapeutic application of pore-forming proteins in cancers

Most conventional anticancer therapies eliminate malignant cells through one of three mechanisms: inducing cell differentiation and thus pushing malignant cells into a non-proliferative, differentiated state; triggering cellular stress that renders cancer cells nonviable; or inhibiting upstream anti-apoptotic mediators such as growth factor receptors and signaling kinases to reduce apoptosis resistance and render tumor cells more sensitive to oncogenic and environmental stress (e.g. hypoxia). While these strategies can be effective, they are often associated with high non-specific toxicity, and cancer cells frequently evolve mechanisms to evade them. In contrast, the superior efficacy of therapeutic immune cells lies in the nature of the cytotoxic molecules they deploy. These molecules can directly activate the terminal, effector stages of regulated cell death (RCD) pathways, offering cancer cells little opportunity to develop resistance. Among the most potent of these cytotoxic agents are pore-forming proteins (PFPs). The ability of PFPs to directly perforate membranes and lyse cancer cells makes them compelling candidates for the development of novel and effective cancer therapies. Compared to conventional treatment modalities such as chemotherapy, radiotherapy, and immunotherapy, PFP-based approaches offer a unique advantage: potent, targeted elimination of malignant cells with reduced collateral damage to healthy tissues.

In this context, nanotechnology offers a powerful tool for enhancing the therapeutic utility of PFPs. Smart, biocompatible nanoparticles can be engineered to precisely deliver extracellular-acting PFPs (e.g., PRF-1 and granulysin) directly to tumor sites or to regulate the expression and activation of intracellular PFPs (e.g., GSDMs and MLKL). The following section reviews recent advances in the therapeutic application of both secreted and intracellular PFPs, highlighting their translational potential in the context of cancer treatment.

### Perforin-1

Despite the potency of PFPs, not every interaction between cytotoxic lymphocytes and their targets results in cell death. For example, cancer cells can modify their membrane composition to resist PRF1-mediated lysis by altering their composition, such as increasing lipid order and membrane stiffness by increasing the cholesterol content or by expressing PS in the outer membrane leaflet to make the membrane surface negatively charged [213, 214]. Consequently, reducing membrane stiffness or lipid order through depletion of cholesterol could enhance the effectiveness of PRF1. It has been demonstrated that intratumoral injection of the cholesterol-depleting agent, methyl-β-cyclodextrin (MeβCD; 5 mM for 30 min) can increase T-cell cytotoxicity, while having negligible impact on tumor-infiltrating T cells due to its transient activity [215]. Cholesterol oxidase (COD) is another molecule for cholesterol depletion. When administered in combination with a metal–organic framework (MOF) consisting of hafnium and 5,10,15,20-tetra(p-benzoato)porphyrin (denoted as Hf-TBP/COD), it did not only induce cholesterol depletion from the phospholipid bilayer, but also generated ROS through Hf-TBP in response to light-irradiation (660 nm, 100 mW cm^−2^). This combination therapy repressed the immuno-suppressive properties of TME and increased T cell infiltration [216].

However, the challenges are not limited to the cancer cells’ membrane; they also arise from the insufficient activation of cytotoxic lymphocytes, leading to low concentrations of PRF1 released into the IS. To address this issue, Zhao and colleagues prepared CD63-functionalized nanocarriers based on zeolitic imidazolate framework-8 (ZIF-8) for the targeted delivery of PRF1 and GZMB into lysosomes of T cells. In a 4T1 mouse tumor xenograft assay, T cells loaded with nanocarrier-delivered PRF1 and GZMB released greater amounts of these cytotoxic effectors compared to non-loaded T cells, which enhanced tumor cell apoptosis [217].

Despite these initial results, in vivo overexpression of PRF1 in immune cells poses a risk of systemic adverse effects. In light of this challenge, PRF1 expression was introduced into target cells directly, via a liposomal nanocarrier encapsulating a PRF1 expression vector driven by the prostate-specific antigen (PSA) promoter, so PRF1 expression was restricted to the prostate cancer cells. Accordingly, elevated PRF1 levels were detectable in the tumor, offering a novel therapeutic strategy for the treatment of advanced prostate cancer [218]. Moreover, the efficient penetration of nanocarriers deep into the TME is a critical factor that need to be considered. A recent study has demonstrated that functionalizing porous polymeric nanoparticles encapsulating PRF1 and GZMB with MMP-2 responsive peptides may achieve this goal. Since the TME of many tumors contains high levels of MMP-2, this system facilitates the targeted release of the cytotoxic cargo directly in the TME where they can disperse in the tumor stroma and reach multiple cancer cells. Indicating its potential, the nano-delivery platform showed strong anti-tumor activity in 4T1 tumor-bearing mice compared to those treated with GZMB or PRF1 alone [219].

### Granulysin

As previously discussed, the 9-kDa processed form of granulysin is a cytotoxic effector molecule stored in the LGs of cytotoxic lymphocytes and has cytotoxic activity against both microbes and some tumor cells. Thus, its therapeutic potential for cancer therapy has been tested through various strategies. One notable approach involved intratumour injection of recombinant granulysin, in an in vivo model of multiple myeloma [220]. The study highlighted two major challenges: the reliance on intratumoral injection and the lack of tumor specificity of recombinant granulysin. To overcome these limitations, advanced targeted therapeutic strategies, including immunotoxin formulations and nano-delivery platforms may hold the key for the therapeutic use of granulysin.

The first strategy is the use of immunotoxins, which combine a cytotoxic agent, such as granulysin, with an antibody as a targeting moiety to achieve tumor-specific delivery. In this context, Ibáñez-Pérez and colleagues engineered a granulysin-based immunotoxin by fusing the granulysin gene to a targeting antibody fragment (MFE23) that specifically recognizes carcinoembryonic antigen (CEA). Their study systematically evaluated the efficacy of this granulysin-based immunotoxin, MFE23GRNLY, in HeLa-CEA tumor-bearing mice, providing the first proof-of-concept for its use in cancer therapy [221]. However, the application of CEA-targeting immunotoxins is limited by the restricted expression of CEA to colorectal and gastric cancers. To address this challenge, Guerrero-Ochoa and colleagues optimized a granulysin-based immunotoxin by targeting the Tn antigen, a marker of aberrant glycosylation, present in a wide range of tumor types. One well known protein presenting such aberrant glycosylation is the ECM protein, mucin-1. The study used the SM3 antibody targeting aberrantly glycosylated mucin-1 (Tn-Muc1) and conjugated it with granulysin (SM3GRNLY). SM3GRNLY offered several advantages, including high production yields, specific tumor targeting across diverse cancer types, and low immunogenicity, making it a promising therapeutic approach for cancer treatment [222]. This group later developed another immunotoxin, AR20.5GRNLY, also targeting the MUC-1-Tn antigen. Antigen affinity, in vitro cytotoxicity, and in vivo anti-tumor efficacy were assessed, and both immunotoxins demonstrated potent toxicity against pancreatic adenocarcinoma tumor-bearing mice through inducing apoptosis, necroptosis, and necrosis [223].

The second approach to increase therapeutic efficacy of recombinant granulysin is the use of nanocarriers. Nanotechnology provides a wide variety of targeted and designable nanoplatforms that carry therapeutic agents, protect them from premature degradation, and deliver them to the desired location [224]. Liposomes are regarded as promising and versatile nano-formulations, capable of improving the efficacy of encapsulated cargos [225]. A recent study has demonstrated that the cytotoxicity of recombinant granulysin was markedly improved when it was conjugated to a liposome [226]. Moreover, nanocarriers can be also designed as smart systems that respond to internal or external stimuli, enabling the controlled release of granulysin at the desired site [227]. Compared to the first strategy, employing nanoplatforms might be a more potent approach for addressing the limitations associated with granulysin-based immunotoxins.

### Gasdermins

GSDMs play a dual role in cancer therapy due to their inconsistent expression profiles in various cancers. For example, GSDMA, GSDMC, and GSDMD are underexpressed in esophageal and gastric cancers, while GSDMB is overexpressed and acts as an oncogene in these cancers [228]. In contrast, GSDME appears to be a tumor suppressor gene, with its expression silenced by promoter methylation in gastric- [229], breast- [230] and colorectal cancers [231]. Also, while GSDME expression is higher in esophageal squamous cell carcinoma (ESCC) compared to the normal tissue, its overexpression is associated with better prognosis [232]. Similarly, elevated GSDMD expression in endometrial cancer correlates with improved anti-tumor immune responses and a more favorable prognosis [233]. GSDMC, while highly expressed in pancreatic ductal adenocarcinoma (PDAC), is linked to cancer stemness and metastasis [234]. These findings highlight the importance of evaluating GSDM expression patterns in tumors compared to normal tissues and exploring their relationships with immune factors to clarify their roles as tumor suppressors or tumor promoters. This could inform new therapeutic strategies focusing on the re-expression or inhibition of GSDMs.

Therapeutic modalities such as anticancer drugs, photodynamic therapy (PDT), photothermal therapy (PTT), and RT can induce pyroptosis by cleaving GSDM proteins in tumor cells. For example, paclitaxel and cisplatin trigger pyroptosis via the caspase-3/GSDME pathway in A549 lung cancer cells [235]. However, cisplatin also upregulates cyclooxygenase-2 (COX-2), contributing to drug resistance and an immunosuppressive TME. To overcome this, combination therapies and engineered nanosystems can selectively induce pyroptosis in cancer cells while minimizing off-target effects. To this end, Yu and colleagues developed a nanopolymer that delivers both a COX-2 inhibitor and anti-cancer agents to pancreatic cancer cells, reducing drug resistance and promoting pyroptosis [236]. In an alternative approach, arsenic trioxide (As₂O₃) encapsulated in a nanoplatform was used to reduce DNA methyltransferase expression, thus enhancing GSDME-mediated pyroptosis in hepatocellular carcinoma [237].

PDT is an emerging treatment that uses photosensitizers (PSs) to generate reactive oxygen species (ROS) upon light exposure. IR700DX-6T, a mitochondria-targeted PS, induces GSDME-dependent pyroptosis in colorectal cancer cells. Combined with decitabine (a DNA methyltransferase inhibitor) and anti-PD-1 antibodies, this approach reversed GSDME silencing and improved therapeutic efficacy [238].

PDT combined with PTT offers a non-invasive strategy for inducing pyroptosis. For instance, when IR780, a photosensitizer was delivered via nanomicelles to CD320-overexpressing gastric cancer cells, upon near-infrared (NIR) light exposure mitochondrial ROS production increased, activating the NLRP3 inflammasome, caspase-1, and GSDMD, leading to pyroptosis and enhanced anti-tumor immunity [239].

RT is another therapeutic approach capable of inducing pyroptosis in esophageal carcinoma cells through caspase-3/GSDME pathway [240]. To utilize this effect of RT, therapies targeting GSDMs to induce pyroptosis using plasmids, microRNA, and CRISPR/Cas9 technology have garnered significant attention. For example, Yin and colleagues have developed an ultrasound (US)-controlled perforation system (UPS) to deliver a plasmid to 4T1 breast cancer cells. This system allowed plasmid entry into cells to express GSDME and making the cells more sensitive to low doses of X-ray irradiation [241]. Recent advances in GSDM-targeted therapies are summarized in Table 2.

**Table 2:** The most recent strategies targeting GSDM expression

GSDM	Type of cancer	Relative expression of GSDM	Therapeutic approaches	Outcome	References
GSDMD	Colon cancer	Decrease	Restoring GSDMD expression Nanoplatform: Calcium carbonate nanoparticles functionalized with hyaluronic acid Cargo: Plasmid encoding full length GSDMD protein	The prepared nanoplatform induces tumor-specific pyroptosis, promotes immune memory effects, and prevents tumor recurrence	[242]
GSDMB	Breast cancer	Increase	Targeting intracellular GSDMB oncoprotein Nanoparticle: Hyaluronic acid nanocapsule Cargo: Anti-GSDMB antibody	Anti-GSDMB nanotherapy effectively reduce tumor growth, metastatic behavior, and drug resistance in HER2 positive breast cancer cells	[243]
	Breast cancer Melanoma	Decrease	Restoring GSDMB expression Nanoparticle: Cationic lipid nanoparticles termed as AA3-Dlin LNPs Cargo: Single-agent mRNA coding the N-terminus of GSDMB	Nanoparticles effectively deliver the N-terminus of GSDMB to trigger the pyroptosis pathway without caspase cleavage in both models. The prepared platform provides strong immunity by reprogramming the TME from a cold to a hot state	[244]
GSDME	Breast cancer	Decrease	Activation of GSDME using antagomir Treatment: Cetuximab and miR-155-5p antagomir	The combination of cetuximab with the miR-155-5p antagomir effectively promoted pyroptosis in triple-negative breast cancer cells	[245]
	Colon cancer	Decrease	Activation of GSDME Nanoplatform: Lipid‐coated PLGA nanoparticles Cargoes: GSDME expressing plasmid DNA with a heat‐inducible promoter HSP70 and a photosensitizer indocyanine green (ICG) Alternative therapeutic approaches: Chemotherapy (Oxaliplatin) and photothermal therapy (PTT)	The nanoplatform efficiently delivers therapeutic cargoes, and upon irradiation, ICG generates localized hyperthermia, further promoting GSDME expression. Concurrently, oxaliplatin activates caspase-3, triggering GSDME cleavage and inducing pyroptosis	[246]
	Breast cancer	Decrease	Restoring GSDME expression Nanoplatform: Dual-layer polydopamine hybrid nanoplatform (DMP@P) Cargoes: and DNA methyltransferase inhibitor decitabine (DCT) and mitoxantrone (MIT) Alternative therapeutic approach: PTT	PDA absorbs near-infrared (NIR) laser irradiation, increasing Ca^2^⁺ influx and caspase-3 activation, ultimately resulting in GSDME cleavage. Additionally, MIT activates caspase-3, while DCT enhances GSDME expression, collectively leading to robust pyroptosis in breast cancer	[247]
	Melanoma	Decrease	Triggering endogenous GDSME expression Nanoplatform: Nano-CD (a polymer type) Cargoes: CRISPR/dCas9 Plasmid (for GSDME expression) and cisplatin Alternative therapeutic approach: Immune checkpoint blockade	Nano-CD delivers both cargoes, leading to the synthesis of endogenous GSDME protein, which is subsequently cleaved by cisplatin-induced activated caspase-3. Nano-CD, in combination with a PD-1 antibody, provides strong immune memory, preventing relapse and lung metastasis in mouse melanoma models	[248]
	Osteosarcoma	Decrease	Upregulation of GSDME protein levels Vector: LPAD contain ethanolamine (EA), ethylenediamine (ED) and poly (glycidyl methacrylate) Cargo: GSDME plasmid Alternative therapeutic approaches: Chemotherapy (Cisplatin)	The proposed combination therapy induces the expression of GSDME, promoting pyroptosis and reshaping osteosarcoma microenvironment by transforming its cold into the favorable hot feature	[249]

### MLKL

Genetic and epigenetic changes in necroptosis pathways have been identified in many tumor types. For instance, diminished MLKL expression have been documented in ovarian- [250], cervical- [251], gastric- [252] and colon cancers [253], correlating with poor overall survival. To overcome this, the Saelens group employed three distinct strategies to restore MLKL expression in melanoma and colon cancers [254–256].

In the first method, MLKL mRNA was delivered into melanoma and colon carcinoma tumors using electroporation, combined with the immune checkpoint inhibitor blocker, anti-PD1. This combination therapy resulted in a remarkable induction of neo-epitope-specific T cell responses, providing protection against the growth of both primary and distal tumors [256]. Secondly, exogenous recombinant MLKL protein was delivered into murine B16 melanoma tumor cells using laser-induced vapor nanobubble (VNB) photoporation. In this approach, B16 tumor cells were incubated with gold nanoparticles, which rapidly heat up upon laser irradiation, causing the evaporation of surrounding water and the formation of vapor nanobubbles. Once the thermal energy is consumed, the nanobubbles collapse, temporarily permeabilizing the cell membrane and facilitating the efficient entry of MLKL [254]. Lastly, vaccinia viruses (VACV) were utilized for the intratumoral delivery of MLKL protein in a B16 melanoma tumor model, resulting in potent anti-tumor activity [255]. Overall, these studies highlighted that inducing necroptosis through the delivery of MLKL mRNA or protein can enhance anti-tumor immunity, emphasizing its potential as a treatment to trigger immunogenic tumor cell death and consequent activation of anti-tumor immune reaction.

Non-viral delivery systems, such as nanocarriers, have demonstrated significant potential for the co-delivery of genetic agents and therapeutic molecules to cancer cells. In this context, Sun and colleagues utilized liposomes for the simultaneous delivery of MLKL plasmid DNA, zVAD (a pan-caspase inhibitor peptide) and second mitochondria-derived activator of caspase (SMAC) to induce necroptosis in colon cancer cells. This approach demonstrated a synergistic interaction between the gene therapy and the drugs, leading to RIPK3-dependent necroptosis in CT26 tumor-bearing BALB/c mice [257]. Recent advancements have introduced a pH-sensitive nanovector-based gene carrier system using highly branched poly(β-amino ester)s (HPAEs) for the delivery of MLKL plasmid DNA. When combined with anti-PD1 therapy, the HPAEs nanovector demonstrated significant potential in remodeling the TME, promoting infiltration of dendritic cells and cytotoxic CD8^+^ T cells into the tumor, thereby inhibiting metastasis in E0771 tumor-bearing mice [258]. A meta-analysis of 613 cancer patients has demonstrated that decreased MLKL expression is correlated with advanced tumor stage and increased lymph node metastasis [259]. At the same time, high MLKL expression in nasopharyngeal carcinoma has been identified as a negative prognostic marker and knockdown or knockout of MLKL in radioresistant nasopharyngeal carcinoma cells impeded metastasis by suppressing epithelial mesenchymal transition [260]. Thus, for future MLKL-based therapies, it is crucial to assess the expression patterns and opposing functions of MLKL across a broad range of cancers before considering its exploitation in anti-cancer therapies.

## Conclusions and future directions

There are a wide variety of PFPs across all domains of life, sharing the common feature of inducing membrane damage. In this review, we classified human PFPs based on their functional properties, distinguishing between secreted PFPs that mediate non-autologous cell lysis and intracellular PFPs that act in a cell-autologous manner, permeabilizing the membranes of the cells from inside.

Increased insights into the function and mechanism of action of PFPs reveal their complementary and specialized functions, ranging from target specificity and diverse biological functions to their interactions with specific types of cell death pathways. For instance, complement system proteins, granulysin and PRF2 target bacterial membranes, PRF1, NINJ-1 and MLKL disrupt the plasma membrane of host cells, while GSDMs can act on both plasma membranes and subcellular compartments. From a functional perspective, PRF1 facilitates the delivery of cytotoxic effectors and regulates ion fluxes, whereas GSDM and MLKL pores play a crucial role in the release of pro-inflammatory mediators and alarmins. Moreover, each PFP is associated with a specific subset of cell death mechanisms. For example, PRF1, through the delivery of GZMs, is involved in apoptosis, while granulysin plays a role in microptosis and GSDMs and MLKL are key triggers of pyroptosis and necroptosis, respectively.

Extensive scientific evidence supports the crucial role and therapeutic potential of PFPs in a number of diseases, especially in cancer. The lytic activity and ability of PFPs to induce membrane distortion represent a promising area for future investigations to develop novel cancer therapies. However, the role of PFPs, particularly the intracellular ones, is often paradoxical, necessitating studies that assess their function in specific cancer types and different molecular pathways. Such investigations are critical to carefully evaluate and design precise therapeutic strategies aimed at re-expressing or knocking out these proteins. Additionally, cancer cells employ resistance mechanisms against PFPs, including the activation of repair pathways to inhibit membrane perforation and the expression of intracellular mediators to neutralize their function. In other cases, the TME suppresses cytotoxic lymphocytes, resulting weak cytotoxic attack where too little extracellular PFPs are released to kill the cancer cells.

New investigations into the biological functions of PFPs are providing deeper insights into the crosstalk between different types of cell death, uncovering potential regulatory mechanisms and therapeutic implications, and offering new perspectives on potential treatment strategies. PANoptosis represents a novel and multifaced inflammatory PCD pathway driven by caspases and RIPKs that simultaneously integrates pyroptosis, apoptosis, and necroptosis, forming a *‘death triangle’* of cells [261]. Targeting PANoptosis-associated PFPs using appropriate strategies emerges great promise for overcoming cancer treatment resistance and improving the effectiveness of current therapies. In the last two decades, nanotechnology has provided vast array of opportunities for cancer immunotherapy. Encapsulation of specific molecules, such as drugs and genetic agents like CRISPR/Cas9, siRNA, and mRNA, into nanocarriers with tumor targeting strategy has led to address PFP resistance and represents promising strategy for effective induction of PANoptosis in specific cancer models.

Despite these advances, several emerging questions remain and must be addressed to fully realize the therapeutic potential of PFPs. These include understanding how PFPs are regulated, identifying strategies for their selective targeting and characterizing the structural plasticity of PFPs that may influence their activity and specificity in different biological contexts. Addressing these challenges will be essential for refining PFP-based therapies and optimizing their application across various disease models.

## Data Availability

No datasets were generated or analysed during the current study.
